# Integrated analysis of the local and systemic changes preceding the development of post-partum cytological endometritis

**DOI:** 10.1186/s12864-015-1967-5

**Published:** 2015-10-19

**Authors:** Cathriona Foley, Aspinas Chapwanya, John J. Callanan, Ronan Whiston, Raúl Miranda-CasoLuengo, Junnan Lu, Wim G. Meijer, David J. Lynn, Cliona O’ Farrelly, Kieran G. Meade

**Affiliations:** Animal & Bioscience Research Department, Animal & Grassland Research and Innovation Centre, Teagasc, Grange, Co. Meath, Ireland; Comparative Immunology Group, School of Biochemistry and Immunology, Trinity College, Dublin 2, Ireland; Ross University, School of Veterinary Medicine, St Kitts, P.O. Box 334, Basseterre, West Indies Dominica; UCD School of Veterinary Medicine, University College Dublin, Dublin 4, Ireland; UCD School of Biomolecular and Biomedical Science and UCD Conway Institute of Biomolecular and Biomedical Research. University College Dublin, Dublin 4, Ireland; South Australian Health & Medical Research Institute, North Terrace, Adelaide, 5000 SA Australia; School of Medicine, Flinders University, Bedford Park, Flinders, 5042 SA Australia

## Abstract

**Background:**

The regulation of endometrial inflammation has important consequences for the resumption of bovine fertility postpartum. All cows experience bacterial influx into the uterus after calving; however a significant proportion fail to clear infection leading to the development of cytological endometritis (CE) and compromised fertility. We hypothesised that early immunological changes could not only act as potential prognostic biomarkers for the subsequent development of disease but also shed light on the pathogenesis of endometritis in the postpartum dairy cow.

**Methods:**

Endometrial biopsy RNA was extracted from 15 cows at 7 and 21 days postpartum (DPP), using the Qiagen RNeasy^®^ Plus Mini kit and quality determined using an Agilent 2100 bioanalyser. Disease status was determined by histpathology based on inflammatory cell infiltrate. RNA-seq of both mRNA and miRNA libraries were performed on an Illumina® HiSeq^™^ 2000. Paired reads were aligned to the bovine genome with Bowtie2 and differentially expressed genes were identified using EdgeR. Significantly over-represented Gene Ontology terms were identified using GO-seq, and pathway analysis was performed using KEGG. Quanititative real-time PCR was also performed for validation (ABI 7500 fast). Haematology was assessed using an automated ADVIA 2120 analyser. Serum proteins were evaluated by ELISA and metabolite analysis was performed using a Beckman Coulter AU 400 clinical analyser. Terminal-restriction fragment length polymorphism (T-RFLP) was used to obtain fingerprints of the microbial communities present.

**Results:**

Next-generation sequencing from endometrial biopsies taken at 7 DPP identified significant induction of inflammatory gene expression in all cows. Despite the common inflammatory profile and enrichment of the *Toll-like receptor* and *NFκB* pathways, 73 genes and 31 miRNAs were significantly differentially expressed between healthy cows (HC, *n* = 9) and cows which subsequently developed CE at 7 DPP (*n* = 6, FDR < 0.1). While significant differential expression of 4197 genes in the transcriptome of healthy cows between 7 and 21 DPP showed the transition from a proinflammatory to tissue profliferation and repair, only 31 genes were differentially expressed in cows with CE (FDR < 0.1), indicating the arrest of such a transition. A link betwene the dysregulated inflammatory response and the composition of the uterine microbial communities was suggested by the presence of significant differences in uterine bacterial tRFLP profiles between HC and CE groups. Furthermore, inflammatory activity was not confined to the uterus; decreased circulating granulocytes and increased Acute Phase Protein (SAA and HP) expression levels were detected in plasma at 7 DPP in cows that developed CE.

**Conclusion:**

Our data suggests that the IL1 and IL17 inflammatory cascade activated early postpartum is resolved thereby restoring homeostasis in healthy cows by 21 DPP, but this transition fails to occur in cows which develop CE. Despite a common early inflammatory profile, elevated and differential expression of specific immune genes may identify cows at risk of prolonged inflammation and the development of CE postpartum.

**Electronic supplementary material:**

The online version of this article (doi:10.1186/s12864-015-1967-5) contains supplementary material, which is available to authorized users.

## Background

The complex aetiology of postpartum uterine disease involves multifactorial host and pathogen factors. Occurring at a time of tremendous physiological change as a result of calving, the transition cow is also shifting from a state of immune quiescence that characterises the reproductive tract during pregnancy [1] to heightened immune activation in response to bacterial colonisation of the postpartum uterus. Massive tissue remodelling occurs and the restoration of tissue and immunological homeostasis [2] is necessary before fertilisation can occur and the uterus can support a new pregnancy. In parallel, the transition cow is mobilising tremendous tissue energy reserves to support the change from a non-lactating state to peak milk production.

We and others have demonstrated significant upregulation of several inflammatory mechanisms in the healthy bovine uterus and have suggested that these mechanisms are required for normal involution, tissue remodelling and return of the uterus to a pregnancy-receptive state [3, 4]. Lower proinflammatory gene expression in the early postpartum period may contribute to delayed bacterial clearance and endometritis [5], although higher proinflammatory gene expression (*IL1a*, *IL1b* and *TLR4*) in the first week postpartum has also been reported in cows that developed persistent endometritis [6]. However, the point at which inflammation shifts from physiological to pathological during the development of uterine disease remains to be understood [3, 7].

Anaerobic and aerobic Gram-positive and Gram-negative bacteria are present in the uterus of more than 90 % of cows in the first two weeks postpartum [8, 9], although the complexity of the microbial population and the differentiation between commensal and potentially pathogenic strains is only emerging [10, 11]. Factors governing susceptibility to the development of disease are poorly understood although it is estimated that up to 50 % of dairy cows are affected by some form of vaginal, cervical or uterine disease after calving. The load and species composition of the causitive bacteria will likely play critical roles in driving and also regulating local inflammatory responses within the endometrium. The temporal kinetics and regulation of these changes will influence the efficacy of the immune response, the degree of resultant uterine pathology and the timeframe in which normal reproductive function can be restored.

Manifestations of uterine disease span from clinical metritis or purulent vaginal discharge (PVD), occurring in the immediate postpartum period to sub-clinical or cytological endometritis (CE) which is usually diagnosed from 21 days postpartum (DPP) [12, 13]. Clinical manifestations of uterine disease are associated with prolonged uterine involution, reduced fertility and poorer production [14]. However, the effects of CE on subsequent fertility are not as apparent, which is due to the ability of some cows to resolve sub-clinical infection [15] and also partly accounted for by the various definition of disease used, diagnostic method and time at which disease is assessed. However a meta-analysis of 23 studies found that endometritis increased by 15 the mean number of days a cow was not in calf, decreased the relative risk of pregnancy (at 150 days in milk) by almost one-third and reduced the rate at which cows became pregnant by 16 % [16].

Given the high nutrient demands of the postpartum dairy cow, it is not surprising that recent studies have attributed the development of CE to a physiological dysfunction as a result of inadequate energy supply [15]. However other analysts attribute the rationale for the development of endometritis to high uterine bacterial load and compromised immune function [17]. While there is certainly a genetic component underlying disease susceptibility [18, 19], the interactions between the physiology of the cow, the host immune system and the bacterial challenge will all affect the outcome of uterine infection.

We propose that dysregulated inflammatory responses to local bacterial colonisation early postpartum facilitates the development of endometritis. Following calving, efficient mobilisation of immune cells is required to clear bacterial infection from the uterus, and innate immunity facilitated by polymorphonuclear leukocytes (PMN) is regarded as the predominant mechanism of early defence in the involuting uterus [20]. We have previously demonstrated the induction of specific proinflammatory cytokines and chemokines in cows with inflamed uteri [3, 21], and pan-genomic transcriptomic analysis of uterine biopsies showed a switch from a pro-inflammatory immune response to a tissue remodelling and repair phenotype in healthy beef breed cows [22]. In the present study, we focus on endometrial tissue from dairy cows, with and without CE. Using next-generation sequencing, we have profiled the temporal changes in mRNA gene and miRNA expression at two time points in uterine biopsies with a view to defining differentially expressed inflammatory genes and pathways at 7 DPP which may predict cows at risk of developing uterine disease.

## Results

### Divergent mRNA and miRNA expression profiles at 7 DPP between HC cows and cows that subsequently develop CE

**Fig. 1:**
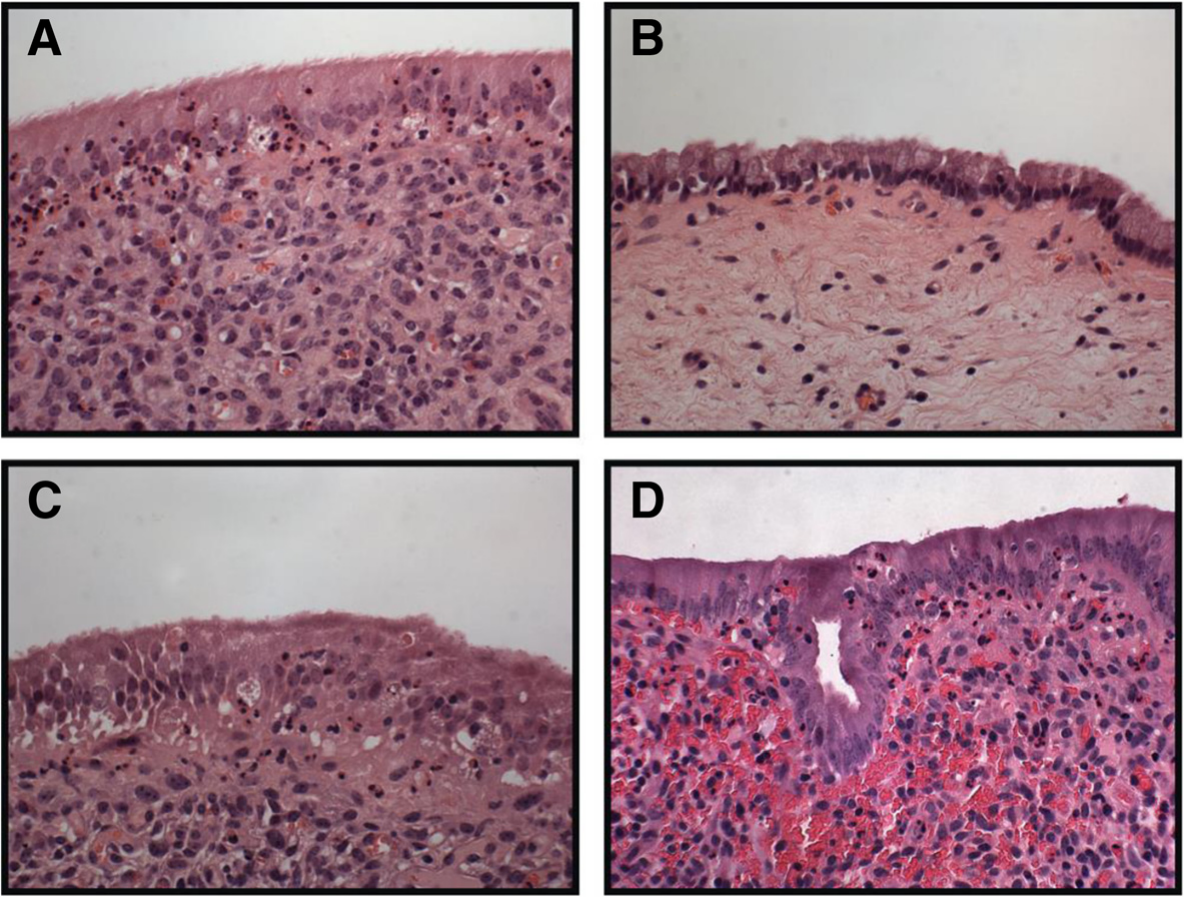
Histological Analysis of Biospies Acccording to Leukocyte Infiltration into the Epithelial and Stromal Layers of the Uterus. **a** and **c** Representative histological classification of endometrial biopsies at 7 DPP shows significant immune cell infiltrate. **b** This influx of leukocytes is not apparent in HC cows, which have resolved inflammation by 21 DPP. **d** Cows that develop CE however, have sustained immune cell infiltrate in the endometrium at 21 DPP. Magnification shown in 400×

**Fig. 2:**
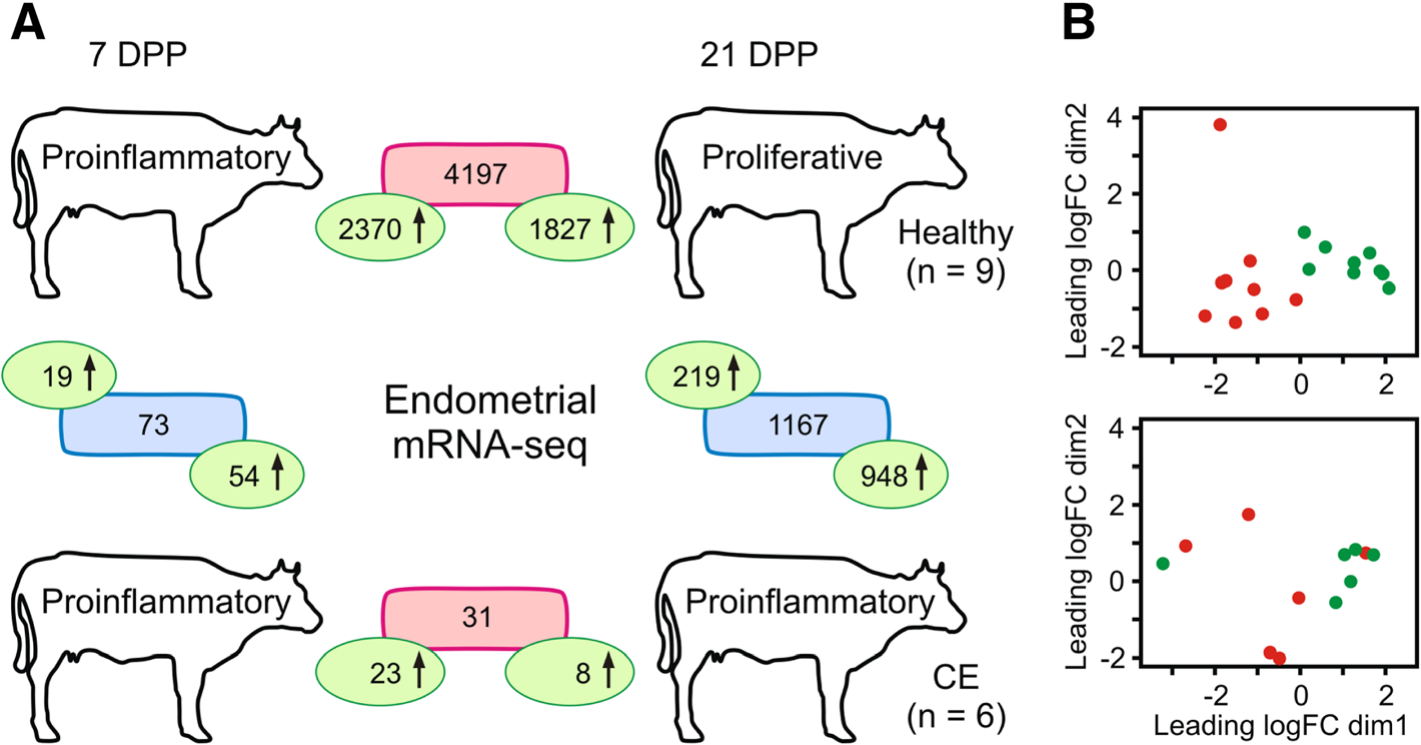
Next-Generation Sequencing (NGS) of messenger RNA from Uterine Biopsies at both 7 and 21 DPP. **a** *top panel* High numbers of genes are differentially expressed in the endometrium from the HC (healthy) group between 7 and 21 DPP, whereas (*bottom panel*) the same transition in gene expression profile between time points is not present in cows that develop CE. The numbers of significantly differentially expressed genes between groups at both postpartum time points are shown (FDR < 0.1). Within group comparisons are shown in red boxes, and between group comparisons are in blue boxes. The numbers in green ovals show the direction in relative gene expression between comparisons. **b** MDS plots generated from endometrial RNA-seq data shows clearer clustering of 7 and 21 DPP time points for the HC group (*top panel*) than for the CE group (*bottom panel*). Nine HC (healthy control) endometrial samples at both 7 DPP (red) and corresponding same animal sample at 21 DPP (green) and **b** six CE (cytologically endometritic) cows at the same two time points are shown. Corresponding gene lists are shown in Additional file 2: Table S2

Uterine biopsies were graded by the degree of immune cell infiltrate into the endometrium at 21 DPP (Fig. 1). Cows yielding low and high scores (0–3 where 3 represents high infiltration of PMN into the epithelial and stromal layers of the endometrium) were assigned to either the healthy control (HC) or cytological endometritis (CE) groups, respectively. Transcriptomic datasets were then generated to assess the differential expression of both genes and miRNA between 7 and 21 DPP. Summary statistics for these 30 mRNA and 20 miRNA libraries are shown in Additional file 1: Table S1. Gene expression results are graphically represented in Fig. 2a and tabulated in Additional file 2: Table S2. Results of bioinformatic analysis of the mRNA and miRNA transcriptomic data are shown in additional supplementary tables (pathway analysis - Additional file 3: Table S3; gene ontology enrichment analysis—Additional file 4: Table S4; DEG miRNA—Additional file 5: Table S5).

Only 73 genes were significantly differentially expressed between the CE and HC animals at 7 DPP, and the majority (54) are increased in expression in the cows that subsequently developed CE (Fig. 2a). A full list of DEG is given in Additional file 2: Table S2 but of the top 10 most significantly DEG, expression of seven genes are increased, and three are decreased in CE at 7 DPP (thereby increased in HC) [Table 1]. Several immune-related genes were differentially increased in CE, the most significant of which was *MASP1* (log_2_ FC 1.9), which encodes an enzyme involved in the lectin pathway of the complement system. The immunoglobulin superfamily member 10 gene (*IGSF10*, log_2_ FC 2.4), interferon gamma-inducible protein 47 gene (*IFI47*) and *MZB1* gene, encoding marginal zone B and B1 cell-specific protein are also significantly increased (log_2_ FC 3.0). In conjunction, increased expression of *CD27*, *CD69*, *CD79A* and *CD79B* all point toward increased B-cell activation in the animals that subsequently develop CE. The latter two genes encode a dimer associated with membrane-bound immunoglobulin in B-cells, thus forming the B-cell antigen receptor (BCR). Furthermore, the *POU2AF1* gene*-* a transcriptional coactivator essential for the response of B-cells to antigens was significantly increased. Seven KEGG pathways were identified as significantly enriched with differentially expressed genes from this dataset (FDR < 0.1). The most significantly enriched pathway identified was *Neuroactive ligand-receptor interaction* and the second was *B-cell receptor signalling* (Additional file 3: Table S3). Of the 19 genes with increased expression in the HC animals (Additional file 2: Table S2), *cytochrome P450, family 26, subfamily A, polypeptide 1* (*CYP26A1*) was the most highly differentially expressed, with a log_2_ FC of 4.7 (Table 1). This gene encodes a monooxygenase, the physiological role of which is to regulate the cellular levels of retinoic acid [23]. Increased expression of the *NPPC* gene was also detected, and this gene encodes natriuretic peptide precursor C which has been shown to stimulate ovarian follicle development [24].

**Table 1:** Top 10 DEG (FDR >0.01) between HC and CE samples at 7 DPP

Gene symbol	Ensembl ID	Gene name	log_2_ FC	FDR
Increased in CE at 7DPP				
*MASP1*	ENSBTAG00000012467	Mannose-associated serine protease 1	1.86	0.001764
*PRSS27*	ENSBTAG00000040394	Protease, serine 27	4.69	0.001764
*TMPRSS11D*	ENSBTAG00000001925	Transmembrane protease, serine 11D	5.02	0.002132
*APOBEC3A*	ENSBTAG00000037800	Apolipoprotein B mRNA editing enzyme, catalytic polypeptide-like 3A	4.10	0.002193
*Unknown*	ENSBTAG00000038520	-	4.74	0.002193
*SLC7A10*	ENSBTAG00000046544	Solute carrier family 7, member 10	3.53	0.002492
*POU2AF1*	ENSBTAG00000006282	POU class 2 associating factor 1	3.67	0.002819
Increased in HC at 7DPP				
*COL4A4*	ENSBTAG00000021310	Collagen, type IV, alpha 4	2.95	0.000950
*CYP26A1*	ENSBTAG00000021118	Cytochrome P450, family 26, subfamily a, polypeptide 1	4.75	0.001764
*NPPC*	ENSBTAG00000003253	Natriuretic peptide C	3.56	0.002193

The expression of 31 miRNAs differentiated between CE and HC animals at 7 DPP, with no endometrial miRNAs being differentially expressed at 21 DPP (FDR < 0.1). Of the differentially expressed miRNA, 26 were increased in CE and five were increased in HC. This number is reduced to 14 and 3, respectively, when a log_2_ FC filter of >1.5 is applied to exclude lowly differentially expressed miRNA (Table 2). Consistent upregulation of the *bta-mir-200* family members was detected in CE endometrial samples, including *bta-mir-200b*, *bta-mir-200c* and *bta-mir-205* (log_2_ FC 7.6). In HC samples, upregulation of *bta-mir-424*, *bta-mir-450b* and *bta-mir-542* was detected.

**Table 2:** DEG miRNA 7DPP between groups (log_2_ FC >1.5)

miRNA name	Ensembl ID	log_2_ FC	FDR
Increased in CE at 7 DPP			
bta-let-7c	ENSBTAG00000029912	1.8	0.050435
bta-mir-31	ENSBTAG00000047544	2.0	0.014096
bta-mir-34b	ENSBTAG00000029856	2.4	0.0257
bta-mir-96	ENSBTAG00000029977	1.7	0.014096
bta-mir-144	ENSBTAG00000029953	1.6	0.064382
bta-mir-147	ENSBTAG00000036401	1.5	0.032079
bta-mir-183	ENSBTAG00000029762	1.5	0.036838
bta-mir-197	ENSBTAG00000029921	2.2	0.000236
bta-mir-200b	ENSBTAG00000030062	1.6	0.036952
bta-mir-200c	ENSBTAG00000029959	2.1	0.012538
bta-mir-205	ENSBTAG00000029854	7.6	6.31E-05
bta-mir-375	ENSBTAG00000029763	3.3	0.000236
bta-mir-451	ENSBTAG00000036414	2.0	0.02058
bta-mir-486	ENSBTAG00000037264	1.9	0.014096
Increased in HC at 7 DPP			
bta-mir-424	ENSBTAG00000036419	2.1	0.012538
bta-mir-450b	ENSBTAG00000043695	1.8	0.043424
bta-mir-542	ENSBTAG00000030024	1.8	0.0257

### Prolonged cytokine signalling, β-defensin and S100 Antimicrobial Peptide expression in CE cows at 21 DPP

At 21 DPP, 1167 genes were significantly differentially expressed between HC and cows diagnosed with CE (Additional file 2: Table S2). The expression of the majority of these genes (948) was increased in expression in the CE animals (Fig. 2a), similar to the 7 DPP results. The most highly differentially expressed gene in cows with CE was Keratin 5 (*KRT5,* log_2_ FC 7.4), which together with the significantly increased *KRT15* and *KRT17* encode keratin proteins involved in the maintenance of the structural integrity of epithelial tissues. The different classes of DE immune genes are shown in Additional file 6: Table S6. These include increased expression of genes encoding cell surface receptors (the TNF-receptor superfamily member *CD27*, the calcium signalling receptor *CD38*, B-cell receptors *CD79A* and *CD79B*); chemokine ligands and receptors (*CCL20*, *CCL22, CCL24* and members of the *CXC* family as well as *CXCR2*); Cytokines and receptors (*IL1R2*, *IL6*, *IL10* and *IL11*); antimicrobial peptides (*DEFB5*, *DEFB7*, *LAP* and multiple members of the S100A gene family) in CE samples at 21 DPP (Additional file 6: Table S6).

Increased expression in HC animals is detected for 219 genes at 21 DPP relative to CE (Fig. 2a). The most highly differentially expressed gene was *TDGF1*, a gene which encodes an epidermal growth factor-related protein (log_2_ FC 5.3). *HIF3A* was also increased, which encodes the alpha-3 subunit of one of several heterodimeric transcription factors that regulate many adaptive responses to hypoxia (Additional file 2: Table S2).

**Fig. 3:**
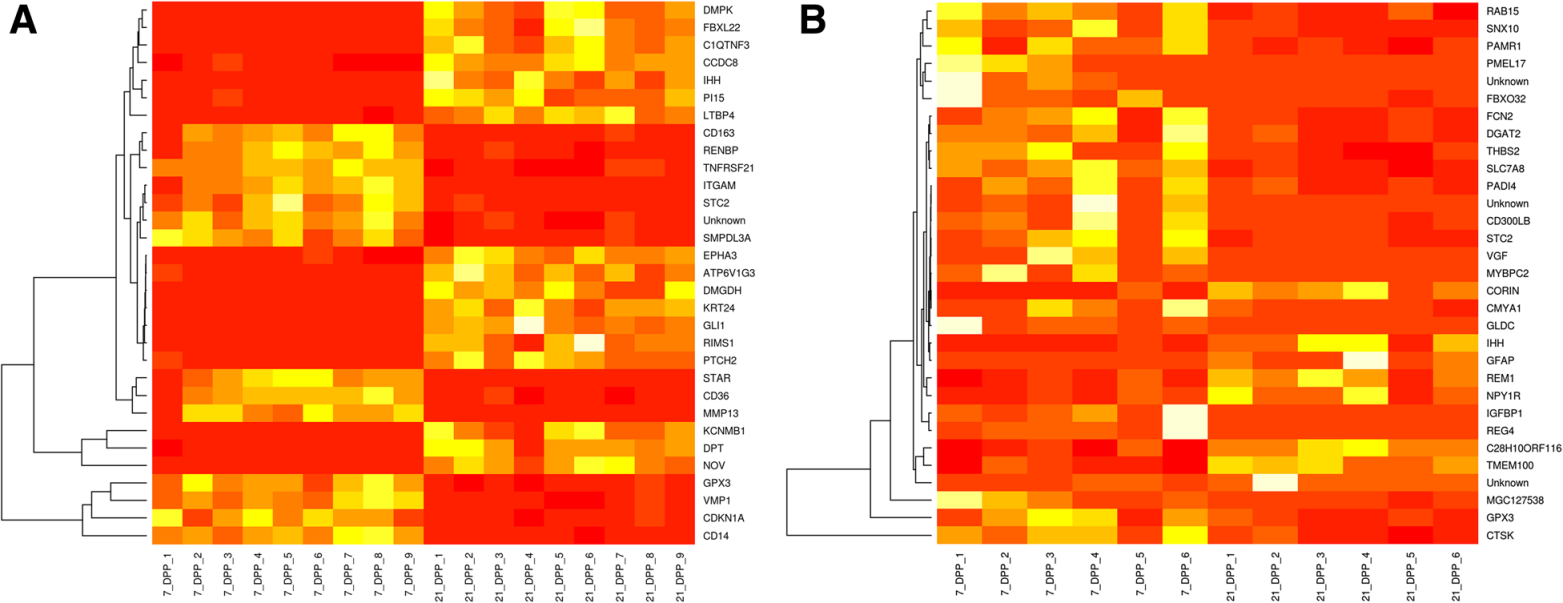
Top 10 Significantly Enriched Biological Processes in the Endometrium Identified by Gene Ontological Analysis. Using significantly differentially expressed gene datasets, gene ontology analysis identifed the enriched biological processes (**a**) in HC cows between 7 and 21 DPP and **b** between HC and CE samples at 21 DPP. The resolution of the inflammatory response in HC cows (**a**) is evident as these defence and innate immune response processes are switched off. The sustained inflammatory response (lack of transition) is evident in (**b**) as these processes are enriched in CE compared to HC at 21 DPP. For full list of enriched gene ontologies, see Additional file 4: Table S4

Forty-five significantly enriched KEGG pathways were identified in this gene dataset of which *Cytokine-cytokine receptor interaction* was the most significant. Multiple other immune-related pathways were also significantly enriched including *B cell receptor*, *Toll-like receptor*, *Jak-STAT*, *NOD-like receptor* and *Chemokine signaling* pathways (Additional file 3: Table S3). The top GO enriched biological process was the *defence response*, followed by *immune* and then *inflammatory response* (Fig. 3b and Additional file 4: Table S4).

### Normal transition from an inflammatory phenotype at 7 DPP to tissue proliferation and repair at 21 DPP does not occur in endometrium of cows with CE

Four thousand one hundred ninety-seven genes were significantly differentially expressed between 7 and 21 DPP samples in HC cows, of which only 483 had a log_2_ FC greater than ±2. Over 100 fold more genes were significantly differentially expressed between 7 and 21 DPP in the HC than was the case for CE animals (Additional file 2: Table S2). Of the 4197 genes, 2370 displayed higher expression at 7 DPP, whereas 1827 were elevated in expression at 21 DPP (Fig. 2a). On the basis of these transcriptomic differences, multidimensional scaling (MDS) plots show the presence of a distinct temporal response profile at 7 and 21 DPP in HC cows. Although some structure is also apparent in the MDS plot from CE animals, the separation is not as clear, which is reflected in the significantly lower numbers of genes differentially expressed between these two timepoints (Fig. 2b). Furthermore, the wide disparity between the 7 DPP CE samples suggests high inter-animal variation in the transcriptional response at this early post-partum time point.

**Fig. 4:**
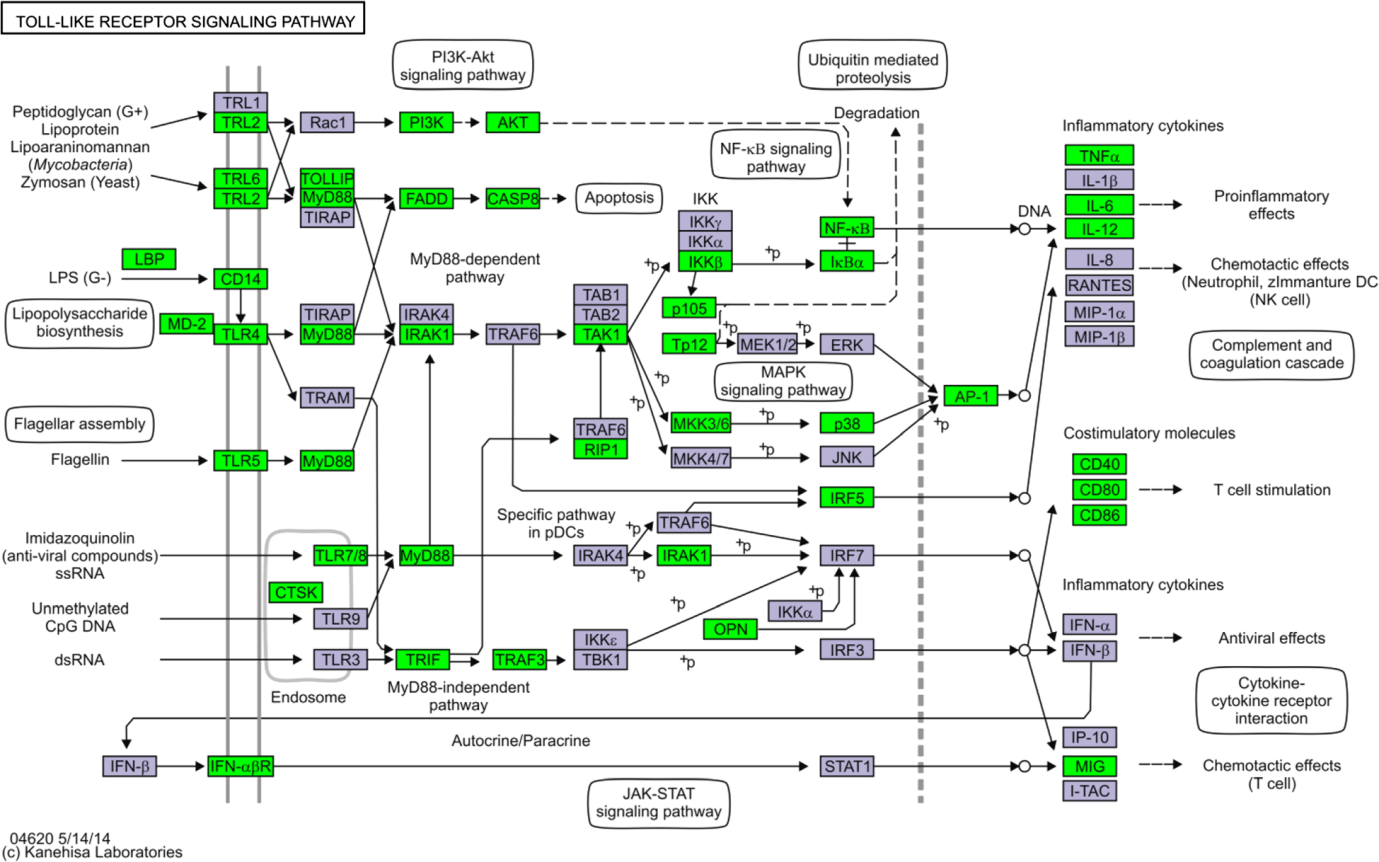
The Kyoto Encyclopaedia of Genes and Genomes (KEGG) annotated the Toll-Like Receptor Signalling Pathway as significantly enriched in the RNA-seq dataset for HC. Green boxes highlight the genes within the pathway that are significantly elevated in expression 7 DPP. For other significantly enriched pathways see Figure S1 (NFkB Signalling Pathway) and Figure S2 (Cytokine-cytokine receptor interaction). For full list of differentially expressed pathways see also Additional file 3: Table S3

The classes of immune genes DE in HC cows between 7 and 21 DPP are shown in Table 3. These include complement proteins involved in antibacterial immunity. Cell surface receptors including *CD14, CD1E* and *CD68*, which are preferentially expressed on monocytes/macrophages were increased. Expression of *CD209* which encodes a transmembrane receptor (also known as DC-SIGN) expressed on the surface of dendritic cells and macrophages, and was also increased at 7 DPP. Genes encoding several receptor signalling and proinflammatory proteins, including the expression of Toll-like receptors (*TLR2*, *TLR4* and *TLR7*), accessory signalling proteins (*CARD4*, *CARD9* and *NLRP1*), and other signalling intermediates were also upregulated. KEGG pathway analysis identified an enrichment of genes from the TLR signalling pathway (Fig. 4). The most highly differentially expressed gene at 7 DPP was IL6 (Table 3). However *IL1A*, *IL1R1* and *IL1R2* as well as *TNF* were also increased in expression. Of particular relevance in the defence against pathogenic bacteria, expression of the antimicrobial peptides, β-defensin expression was significantly elevated at 7 DPP, including *DEFB*, *DEFB5* and *DEFB300*. The expression of the related S100 family of AMPs was also elevated at 7 DPP, including *S100A2*, *S100A5, S100A8, S100A9* and *S100A12*. Genes encoding the acute phase proteins *HP*, lipopolysaccharide binding protein (*LBP*), *SAA1* and *SAA3*, including the mammary associated form (*M-SAA3*) and Transferrin (*TF*) were also increased (Table 3), showing endometrial expression of these liver-associated molecules.

**Table 3:** Significantly DEG (Log_2_ FC >1.3, FDR >0.1) in HC samples between 7 and 21 DPP

Gene symbol	Ensembl ID	Gene name	log_2_ FC	FDR
Complement proteins				
*C1RL*	ENSBTAG00000016204	Complement component 1, r subcomponent-like	1.69	0.000165996
*C2*	ENSBTAG00000007450	Complement component 2	2.67	2.08E-11
*C3*	ENSBTAG00000017280	Complement component 2	1.94	0.002331252
*C3AR1*	ENSBTAG00000019741	Complement component 3a receptor 1	2.25	7.41E-08
*C4BPA*	ENSBTAG00000009876	Complement component 4 binding protein, alpha	3.10	0.002072385
*C5AR1*	ENSBTAG00000020872	Complement component 5a receptor 1	2.87	3.30E-12
*C9*	ENSBTAG00000016149	Complement component 9	1.94	0.094234244
Cell surface receptors				
*CD1E*	ENSBTAG00000009421	CD1e molecule	2.02	9.19E-07
*CD14*	ENSBTAG00000015032	CD14 molecule	3.16	8.72E-26
*CD36*	ENSBTAG00000017866	CD36 molecule	2.43	5.47E-15
*CD40*	ENSBTAG00000020736	CD40 molecule	1.53	0.000643093
*CD68*	ENSBTAG00000000133	CD68 molecule	2.79	3.49E-12
*CD83*	ENSBTAG00000031430	CD83 molecule	1.53	4.91E-05
*CD84*	ENSBTAG00000019033	CD84 molecule	2.23	5.98E-06
*CD86*	ENSBTAG00000013118	CD86 molecule	2.54	3.55E-11
*CD163*	ENSBTAG00000019669	CD163 molecule	3.37	6.04E-13
*CD200R1*	ENSBTAG00000001235	CD200 receptor 1	1.68	0.000161784
*CD209*	ENSBTAG00000007312	CD209 molecule	2.11	0.000602415
*CD300E*	ENSBTAG00000004690	CD300e molecule	2.51	6.68E-08
Pathogen recognition receptors				
*CARD9*	ENSBTAG00000006572	Caspase recruitment domain family, member 9	1.75	9.21E-07
*CARD14*	ENSBTAG00000015261	Caspase recruitment domain family, member 14	1.63	1.05E-05
*TLR2*	ENSBTAG00000008008	Toll-like receptor 2	2.51	1.52E-10
*TLR4*	ENSBTAG00000006240	Toll-like receptor 4	1.38	5.54E-08
*TLR7*	ENSBTAG00000022161	Toll-like receptor 7	2.41	3.28E-07
*NLRP1*	ENSBTAG00000020433	NLR family, pyrin domain containing 1	1.68	2.14E-06
Chemokine ligands and receptors				
*CCL2*	ENSBTAG00000037811	Chemokine (C-C motif) ligand 2	2.20	0.000317988
*CCL8*	ENSBTAG00000014113	Chemokine (C-C motif) ligand 8	2.03	0.003473125
*CCL20*	ENSBTAG00000021326	Chemokine (C-C motif) ligand 20	2.25	0.031650056
*CCR1*	ENSBTAG00000019428	Chemokine (C-C motif) receptor 1	2.65	2.78E-10
*CXCL3*	ENSBTAG00000037778	Chemokine (C-X-C motif) ligand 3	2.52	0.000160609
*CXCL6*	ENSBTAG00000009812	Chemokine (C-X-C motif) ligand 6	3.28	0.001050477
*CXCL9*	ENSBTAG00000038639	Chemokine (C-X-C motif) ligand 9	1.95	0.011169775
*CXCR2*	ENSBTAG00000026753	Chemokine (C-X-C motif) receptor 2	2.62	0.032556379
*CXCL14*	ENSBTAG00000006694	Chemokine (C-X-C motif) ligand 14	1.89	0.000337958
*CXCL17*	ENSBTAG00000018652	Chemokine (C-X-C motif) ligand 17	2.82	0.008009971
Cytokines and receptors				
*IL1A*	ENSBTAG00000010349	Interleukin 1 alpha	2.66	0.016846664
*IL1R1*	ENSBTAG00000005273	Interleukin 1 receptor, type 1	1.59	0.00014519
*IL1R2*	ENSBTAG00000006343	Interleukin 1 receptor, type 2	4.85	3.97E-07
*IL1RL1*	ENSBTAG00000018571	Interleukin 1 receptor-like 1	4.33	0.000262878
*IL6*	ENSBTAG00000014921	Interleukin 6	5.88	2.78E-06
*IL10RA*	ENSBTAG00000005215	Interleukin 10 receptor, alpha	1.73	9.19E-06
*IL18R1*	ENSBTAG00000001034	Interleukin 18 receptor 1	1.85	0.002233603
*IL22RA1*	ENSBTAG00000001100	Interleukin 22 receptor, alpha 1	2.35	7.33E-06
*TNF*	ENSBTAG00000025471	Tumor necrosis factor	1.43	0.007893141
Antimicrobial peptides				
*DEFB*	ENSBTAG00000045649	Defensin, beta	2.33	0.008979282
*DEFB5*	ENSBTAG00000034954	Defensin, beta 5	2.63	0.000491966
*DEFB300*	ENSBTAG00000045626	Beta-defensin 103B-like	2.96	0.028072666
*S100A2*	ENSBTAG00000037651	S100 calcium binding protein A2	2.19	1.75E-06
*S100A5*	ENSBTAG00000000644	S100 calcium binding protein A5	3.22	1.07E-12
*S100A8*	ENSBTAG00000012640	S100 calcium binding protein A8	2.99	0.00064399
*S100A9*	ENSBTAG00000006505	S100 calcium binding protein A9	3.46	7.83E-05
*S100A12*	ENSBTAG00000012638	S100 calcium binding protein A12	2.99	4.98E-05
Acute phase proteins				
*HP*	ENSBTAG00000006354	Haptoglobin	2.40	0.041554479
*LBP*	ENSBTAG00000016864	Lipopolysaccharide binding protein	2.92	0.000384619
*M-SAA3.2*	ENSBTAG00000010433	Mammary serum amyloid A3.2	1.44	0.012854981
*SAA1*	ENSBTAG00000022394	Serum amyloid A1	3.67	0.000142222
*SAA3*	ENSBTAG00000022396	Serum amyloid A3	1.99	0.002751998
*TF*	ENSBTAG00000007273	Transferrin	3.12	1.56E-12

Between 7 and 21 DPP in HC, KEGG pathway analysis identified significant enrichment of 53 pathways with predominant enrichment of immune-related pathways. The top differentially expressed pathway was *Lysosome*, which is the cellular organelle involved in the digestion and removal of bacteria (Additional file 3: Table S3). *Complement and coagulation cascades* was the second most significantly enriched pathway. *Cytokine-cytokine receptor interaction* and *Chemokine signaling*, *Toll-like receptor signaling*, *B cell receptor signaling*, *NOD-like receptor signaling*, *Natural killer cell mediated cytotoxicity* and *MAPK signaling pathways* were all identified as significantly enriched (Additional file 3: Table S3). Gene ontology analysis also identified an enrichment of genes involved with the biological process of the *inflammatory* and *innate immune response* (Additional file 4: Table S4, Fig. 3a). In contrast, *Calcium signalling* was identified as one of the predominant KEGG pathways activated at 21 DPP (Additional file 3: Table S3) and the *regulation of calcium* was identified as a significantly enriched biological process based on gene ontology analysis (Additional file 4: Table S4). The restoration of homestasis in the healthy postpartum endometrium is marked by a reduction in inflammatory gene expression at 21 DPP. In HC cows, the most highly differentially expressed gene at 21 DPP was the transcription factor HIF3A, which regulates the response to low oxygen (log_2_ FC 5.96), which is in contrast to the low fold changes in the majority of significantly elevated genes at this time point. The previous upregulation of inflammatory genes detected at 7 DPP has been replaced with increased expression of a smaller number of chemokines at 21 DPP including *CCL11*. Upregulation of *CD79A* and *CD79B* was also apparent, and Insulin-like growth factor (IGF2) expression is increased. Despite the reduced expression of other inflammatory genes, the *IL17D* gene and IL17 receptor genes *IL17RB* and *IL17RD* are significantly increased at 21 DPP (Additional file 2: Table S2).

**Fig. 5:**
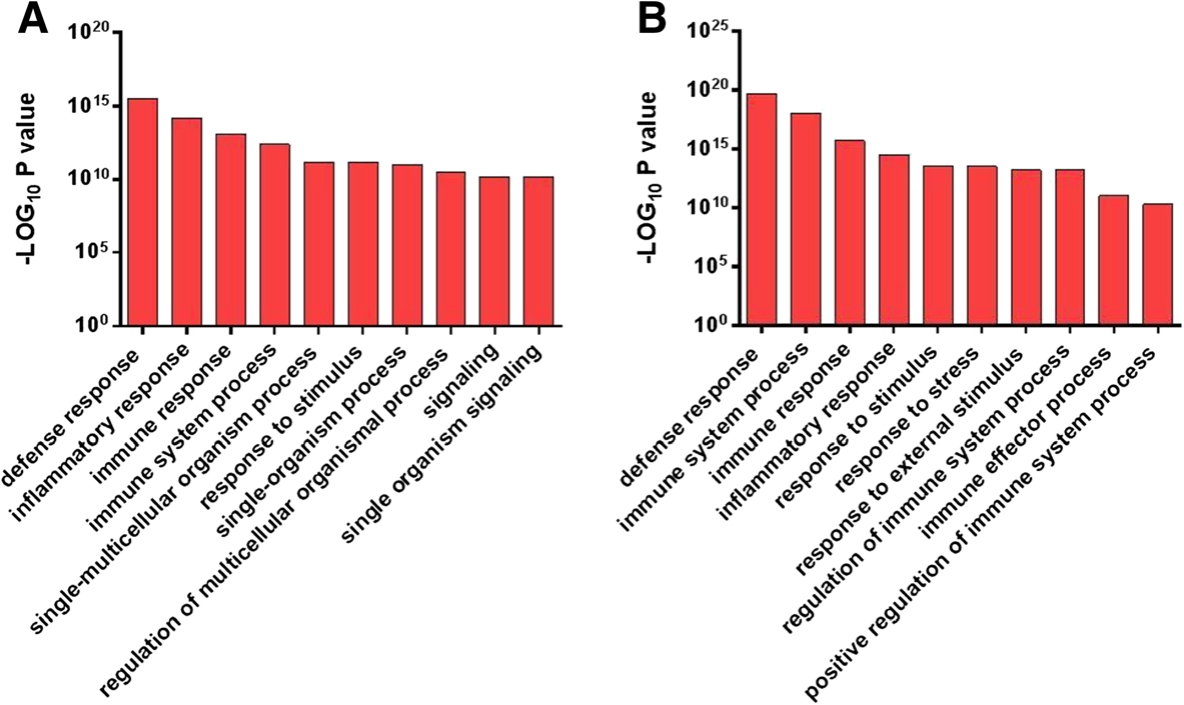
Heat map representation depicting the temporal changes in significantly differentially expressed genes from uterine biopsies between 7 and 21 DPP in the **a** HC and **b** CE cows. All 31 significantly DEGs were used to generate the heatmap in the CE group, and a similar number of the top DEGs (ranked on basis of *P* value) were used for comparative purposes for the HC group. Scale: Yellow indicates high expression and red is low expression. Unsupervised hierarchical clustering dendograms are included for these genes. **a** – DE genes between day 7 and 21 DPP in HC animals and **b** – DE genes between day 7 and 21 DPP in CE cows

In contrast to the marked transition that occurred between 7 DPP and 21 DPP in HC cows, only 31 genes were significantly differentially expressed in the endometrial biopsies from CE cows between the same time points (Additional file 2: Table S2). Heat maps, generated on the basis of these DEG clearly shows two distinct gene sets, which differentiate the 7 and 21 DPP groups. Whereas clear changes in gene expression profile can be seen between samples in the HC group (Fig. 5a), the signal is less clear in the CE group (Fig. 5b). Higher interanimal variation in CE group is also evident in contrast to the clear temporal switch apparent in HC group.

### Validation of inflammatory phenotype in cows with CE

**Fig. 6:**
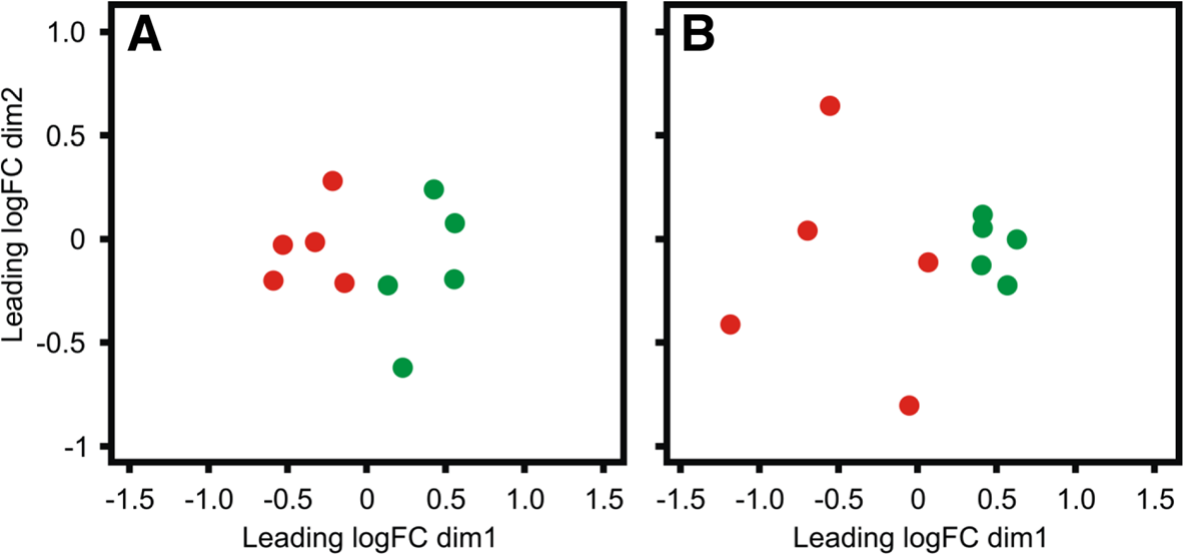
RT-qPCR validation of gene expression changes detected using mRNA-seq. Confirmation of differentially expressed genes from NGS results using quantitative real-time PCR. Significant changes in gene expression of both **a** pro- and anti-inflammatory cytokines as well as other **b** effector molecules of the immune response confirmed the findings from NGS. Results are colour-coded according to comparison and levels of expression of each gene of interest was normalised to expression levels of *PPIA*; D7PP between SCE and HC cows (black), D21PP between SCE and HC (blue), between D7 and D21PP in SCE (red) and between D7 and D21PP in HC (green). Between and within group comparisons are separated by a dotted line. *P < 0.05; **P < 0.01. *n* = 5–8 samples per time point, bars represent mean ± SEM

A panel of 15, predominantly immune-related genes were chosen for validation of the NGS data by qRT-PCR in both HC and CE samples at 7 and 21 DPP. Results showed high concordance in the detection of gene expression changes between NGS and qRT-PCR technologies. Significantly enhanced *IL1B* (FC 18), *IL6* (FC 7.5) and *IL17A* (FC 42) gene expression was evident at 7 DPP in cows that subsequently developed CE (*P* < 0.05, Fig. 6a [black bars]). The presence of a prolonged inflammatory profile in cows that develop CE was supported by significantly higher *IL1A* (FC 25), *IL1B* (FC 41), *IL6* (FC 126), *IL17A* (FC 10) in uterine biopsies from CE cows at 21 DPP relative to HC cow samples (Fig. 6a, [blue bars]). Interestingly, higher expression of *IL1R2* (FC 735) and the gene excoding the anti-inflamammatory cytokine *IL10* (FC 14) was also detected in CE samples at 21 DPP. Although not all genes reached statistical significance, the reduction in the gene expression levels between 7 and 21 DPP is more pronounced for the HC samples (green bars), than the CE cows (Fig. 6a, red bars) supporting the finding of prolonged inflammation at 21 DPP. Significant reductions in the expression of IL1R2 (FC 466) and IL10 (FC 2.8) are detected for HC cows, but are absent for CE cows at 21 DPP (Fig. 6a).

A subset of additional genes differentially expressed using the NGS approach were also validated by qRT-PCR. The significantly different expression of *S100A9*, *IGFBP1*, *SEPINB4*, *DGAT2* and *SAA1/2* support the NGS data (Fig. 6b). Furthermore, validation included genes that were increased in expression in the HC at 21 DPP, including *HIF3A* (P < 0.05, Fig. 6b, green bar). qRT-PCR expression profile for the *CSF3* gene did not correlate with the findings from the NGS data, possibly due to the large inter-animal variation in read-counts detected for this gene (Additional file 2: Table S2).

### Temporal changes in miRNA expression are similar between groups

**Fig. 7:**
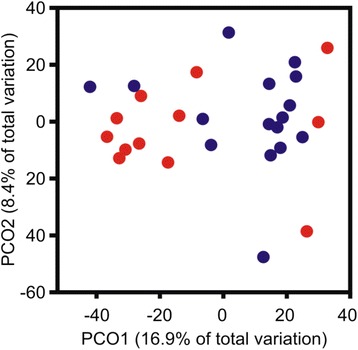
Multi-Dimensional Scaling (MDS) Plots Generated from Endometrial microRNA-seq Data. **a** MDS-plot shown for HC cows at both 7 (red) and 21 DPP (green). Similarly, **b** MDS-plot shown for CE cows at both 7 (red) and 21 DPP (green). Clustering of the D7 and D21 profiles is apparent at both time points for HC and CE cows although tighter clustering of 7 DPP samples for the HC group than for the CE group (**b**) shows a higher degree of variation between samples at 7 DPP (**b**, red). Five HC (healthy control) samples at both 7 DPP (D7, red) and corresponding same animal sample at 21 DPP (D21, green) and B) five CE (cytologically endometritic) cows at the same two time points are shown

Thirty-six miRNA are differentially expressed between 7 DPP and 21 DPP in the HC cows, with 45 in the CE cows (Additional file 5: Table S5). Two miRNA are increased in expression at 7 DPP and 6 are increased at 21 DPP with a log_2_ FC filter of >1.5 in HC (Additional file 7: Table S7.1). Expression of related miRNA family members was apparent with increased expression of *bta-mir-34b* (log_2_ FC 1.7) and *bta-mir-34c* (log_2_ FC 1.6), as well as *bta-mir-449a, bta-mir-449b* and *bta-mir-449c* (log_2_ FC 3.8, 3.4 and 3.9, respectively). Similarly 24 are increased at 7 DPP and 6 increased at 21 DPP with a log_2_ FC filter of >1.5 in CE (Additional file 7: Table S7.2). Related miRNAs *bta-mir-200a, bta-mir-200b* and *bta-mir-200c* are all increased in expression at 7 DPP (log_2_ FC 2.5, 2.5 and 3.2, respectively). Also *bta-mir-205* is the most highly differentially expressed with a log_2_ FC of 7.8. The MDS plot which classifies the animals on the basis of their miRNA expression profile shows a clear separation between 7 DPP and 21 DPP HC animals (Fig. 7a) as well as for the CE animals (Fig. 7b). This structure reflects the numbers of miRNA found to be differentially expressed between timepoints (Additional file 5: Table S5). Interestingly, less clustering is apparent within the 7 DPP CE samples, as was reflected in the mRNA MDS plot (Fig. 2b).

### Distinct endometrial bacterial populations present in CE and HC cows

**Fig. 8:**
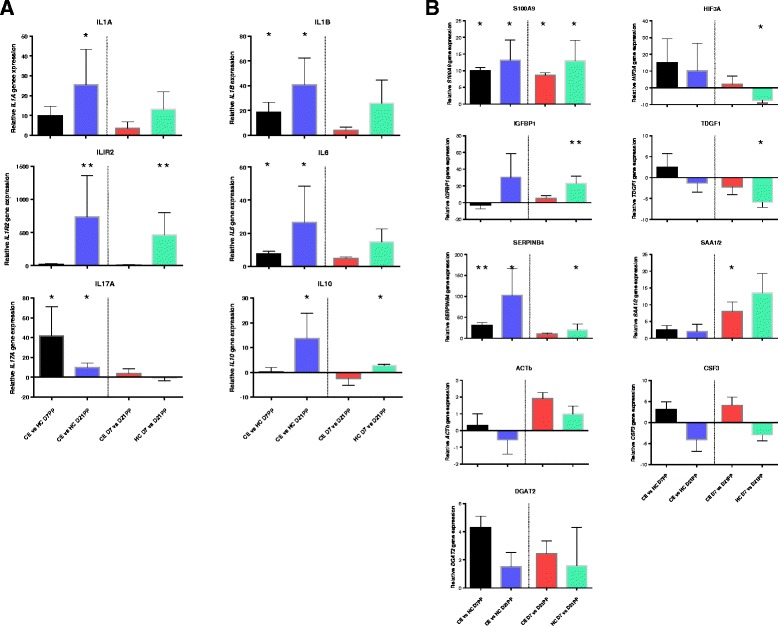
Principal Coordinates Ordination of Endometrial Bacterial Communities. Bacterial community analysis, performed using culture-independent Terminal Restriction Fragment Length Polymorphism (T-RFLP), shows a significant clustering of samples according to their respective microbial communities. HC and CE cows are shown as blue and red circles, respectively

With the aim of determining whether the observed differences in the uterine transcriptomes of CE and HC groups are accompanied by changes of microbial composition, Terminal Restriction Fragment Length Polymorphism (TRFLP) was employed to assess the composition of the microbial communities in the uterus of HC and CE cows at 7 and 21 DPP. In total, 287 operational taxonomic units (OTUs) were observed with a median of 31.5 OTUs per sample. While the median number of OTUs in the CE group was unchanged (25 and 26.5 OTUS at 7 and 21 DPP, respectively), an increase was observed in the HC group, from 29 OTUs to 41.5 OTUs at 7 DPP and 21 DPP, respectively. To correct for the impact of low incidence OTUs, OTUs represented in only one or two samples were removed. This resulted in a data set of 140 OTUs still showing an increased number in the HC group (26 to 35 OTUs vs 23 to 25.5 for the CE group). The ANOVA test revealed significant differences in the number of OTUs (*P* = 0.049) and Tukey’s test attributed these differences to the HC groups at 7 and 21 DPP (*P* =0.048). These results are consistent with an increased bacterial diversity in the HC group during the time frame of the study. The Bray-Curtis similarity was used to compare the microbial communities associated with the samples. Principal Coordinates Ordination (PCO) allowed the visualisation of the distribution of the variation within the data set (Fig. 8). PCO1 and PCO2 accounted for 16.9 and 8.4 % of the variation of the data, in which the first principal coordinate segregated most HC from CE samples. Permanova analysis showed a significant difference in the composition of the microbial communities present between HC and CE cows (*P* =0.049).

### Systemic early cellular and immune changes differentiate between HC and CE cows but metabolite expression levels do not

**Fig. 9:**
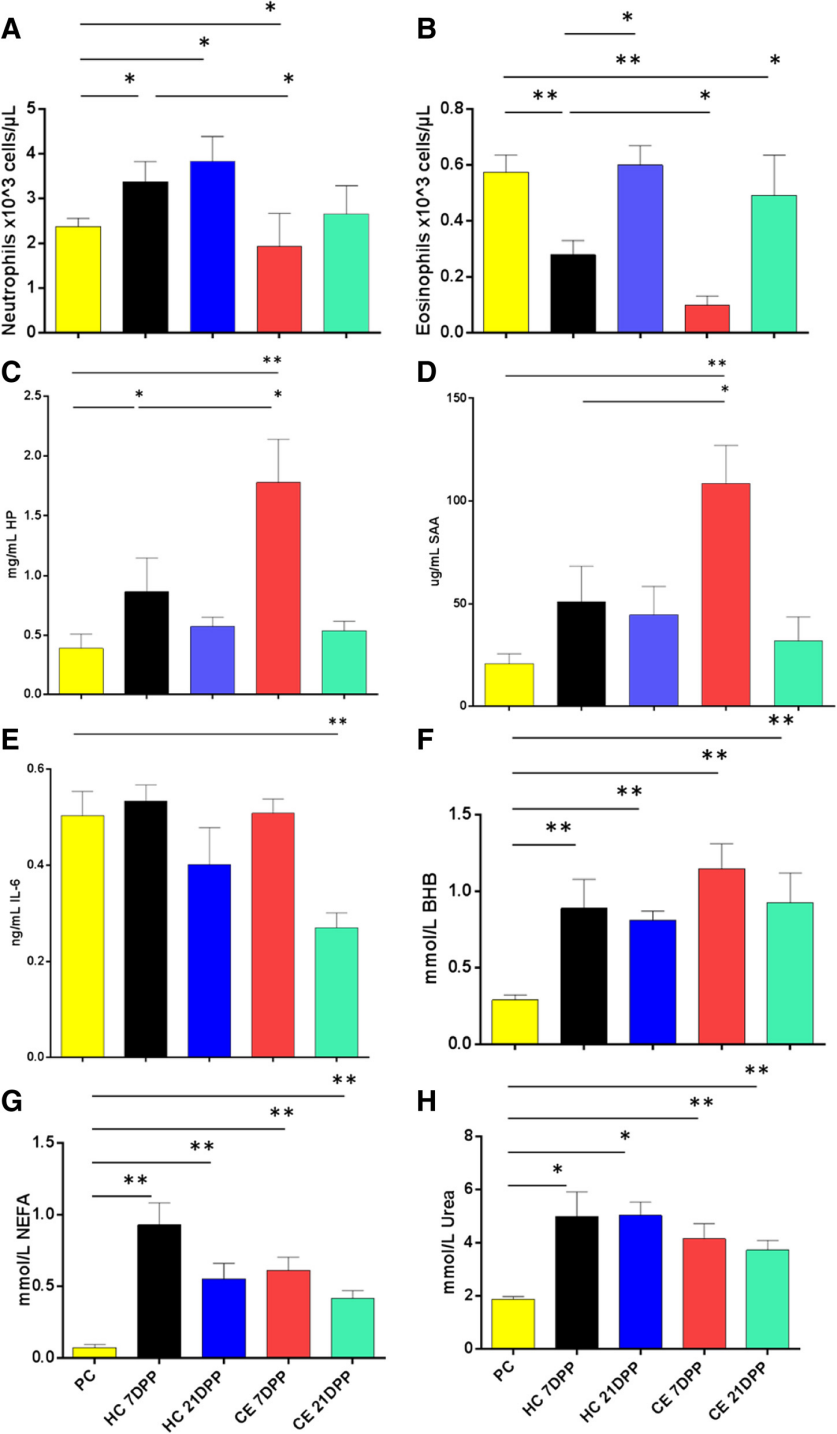
Analysis of Systemic Immune and Metabolite Parameters in HC and CE cows. Significant early reductions in circulating granulocyte numbers in cows that subsequently developed CE. Absolute cell counts in Peripheral blood leukocytes (PBL) for **a** neutrophils and **b** eosinophils. Elevated plasma expression levels for **c** Haptoglobin (HP), **d** Serum Amyloid A (SAA) but not **e** Interleukin 6 (IL-6) were also detected in cows with CE. No significant differences in serum metabolite profile for **f** β-hydroxybutyrate (BHB), **g** non-esterified fatty acids (NEFA) and **h** Urea between cows that developed CE and HC, although temporal changes were significantly changed. Samples labelled as PC (pre-calving), and at 7 and 21 DPP in HC and CE cows.*P < 0.05; **P < 0.01. n = 5–9 samples per timepoint and bars represent mean ± SEM

The numbers of circulating neutrophils in blood decreased significantly from pre-calving values to 1.9 × 10^3^ cells/μL at 7 DPP in blood from cows that developed CE samples (*P* = 0.04) [Fig. 9a]. In contrast the HC animals showed a significant increase in numbers to 3.4 × 10^3^ cells/μL cells at the same time point (*P* = 0.03). The systemic eosinophil counts show even more pronounced changes with a significant reduction in both groups at 7 DPP. In cows with CE, eosinophil numbers dropped to 1 × 10^2^ cells/μL at 7 DPP (*P* = 0.002) and to 3 × 10^2^ cells/μL in HC (*P* = 0.004). By 21 DPP, eosinophil numbers had been restored to pre-calving levels in both groups (Fig. 9b).

In contrast to circulating cell numbers, the concentrations of APPs in serum from CE cows increased significantly at 7 DPP. HP levels increased significantly in both groups from pre-calving levels to 1.8 mg/mL (*P* = 0.007) and 0.9 mg/ml (*P* = 0.03) in CE and HC cows at 7 DPP respectively (Fig. 9c). Expression levels were also significantly different between the HC and CE cows at 7 DPP (*P* = 0.019). Levels of SAA increased from 20.1 μg/mL to 108.1 μg/ml in the CE group at 7 DPP (*P* = 0.002). The elevated SAA expression was over two fold higher than expression levels in HC animals at the same time point (51 μg/ml, *P* = 0.029) [Fig. 9d].

The systemic expression of proinflammatory chemokines (IL-8) and cytokines (IL-1, IL-6, and IL-17) was examined in sera using samples collected at both pre- and post-calving at 7 and 21 DPP. A low concentration of IL8 was expressed and IL8 was not detected in all animals. Average IL-8 expression expression levels were 17 pg/ml at pre-calving and 7 and 21 DPP levels (24 and 26 pg/ml, respectively) were not significantly different. IL-1 and IL-17 cytokines were detected intermittently in serum from some animals, however only IL-6 could be statistically analysed. The only significant difference detected in IL-6 expression levels was a reduction from pre-calving levels in CE animals to 0.29 ng/ml at 21 DPP (*P* = 0.006) [Fig. 9e].

Selected metabolites were also analysed in serum from the same animals, and as expected significant elevation of BHB, NEFA and urea was detected across the calving window (Fig. 9f–h). All three metabolites were significantly increased in both groups at both 7 and 21 DPP relative to levels at calving, but no significant difference between groups was detected. There was no significant change in glucose levels between groups, at any time point (data not shown).

## Discussion

Productive and reproductive efficiency in high yielding dairy cattle requires a disease-free transition period [25] and uterine pathology is a major contributory factor to poorer fertility outcomes [26, 27]. Multiple studies have shown the early activation of an immune response in the postpartum uterus [6, 28] and mechanisms have been proposed to show how bacteria can drive reproductive dysfunction [29]. Using next-generation sequencing of the endometrial transcriptome, our previous work identified the immune transition which occurs in healthy beef-breed cows, during which normal function is restored in the the postpartum period [22]. The current study develops this analysis both in dairy cows, and in cows that were diagnosed with CE. The numbers of differentially expressed genes were over 100 fold higher in the endometrium from healthy cows than in cows with CE, and innate immune genes and signalling pathways were activated in both groups of cows at 7 DPP. Pathway analysis and GO enrichment indicated an abundance of molecules contributing to a proinflammatory environment, including the expression of Toll-like receptors (TLRs), which are key to the early activation of an innate immune response in the endometrium as they detect pathogen associated molecular patterns (PAMPs) on bacteria [30, 31]. In conjunction with NOD/CARD intracytoplasmic proteins, TLRs mediate signals to key transcription factors including NF-κB to activate pro-inflammatory and anti-inflammatory cytokines (IL10) which regulate the innate and adaptive immune response for the efficient clearance of infection.

IL-1 is a key mediator of the host’s inflammatory immune response to infections, and its secretion is elevated during endometrial necrosis [32]. In this study, IL1 expression is enhanced at 7 DPP in cows that subsequently develop CE, and this heightened expression (of both *IL1A* and *IL1B*) is maintained in CE cows at 21 DPP. A high concentration of IL-1 is thought to influence the systemic inflammatory response to induce APP synthesis in the liver, stimulation of IL-6 and the production of neutrophils in the bone marrow [33]. Interestingly, *IL6* is the most highly induced gene in HC at 7 DPP this study. qRT-PCR analysis also confirmed a role for an additional inflammatory cytokine—Interleukin 17 (IL17) at both post-partum time points. *IL17A* gene expression was significantly increased by 42 fold in CE cows relative to HC cows at 7 DPP. IL17A drives the recruitment of inflammatory cells (via increased expression of IL-8) as well as the proliferation of stromal cells which contributes to the pathogenesis of uterine disease in humans [34, 35]. Significantly elevated *IL17A* is also evident in CE samples at 21 DPP. Higher inflammatory gene expression in the first week post-partum is associated with poorer reproductive outcomes across multiple species [6], and it is likely that the excessive inflammation driven by these potent cytokines is associated with the development of CE in cattle. In fact, the significantly increased *IL10* at 21 DPP in the CE samples may reflect an attempt to limit endometrial tissue damage due to excessive inflammation.

Despite the common inflammatory phenotype in HC and CE cows at 7 DPP, two key findings differentiate HC cows from cows that subsequently develop CE. Firstly, transcriptomic profiling identified 73 genes and 31 miRNAs that reliably differentiate the groups at 7 DPP, and as such may hold prognostic potential. Expression profiling has reliably shown that excessive activation of inflammatory cytokines in the early post-partum period may be associated with the subsequent development of uterine disease. However, the early inflammatory environment is not only characterised by the expression of inflammatory cytokines. Expression of cell surface markers (*CD27* and *CD69*) and B cell antigen receptor complex (*CD79a* and *CD79b*) in CE cows are all associated with the activation of B lymphocytes and immunoglobulin synthesis [36]. The transcription factor *POU2AF1* is essential for the response of B-cells to antigens [37] and is highly differentially expressed between HC and CE cows. Based on the suite of differentially expressed genes, KEGG pathway analysis identified the enrichment of B-cell related processes in CE samples. Of relevance is the emerging literature on the role of innate-like B cells in inflammatory diseases [38], which may prove a productive research avenue in relation to endometritis in cattle, but on which little is currently known. Investigation of these markers in a larger panel of post-partum cows is now warranted.

Secondly, the development of CE is characterised by a lack of resolution of inflammation between 7 and 21 DPP. NGS data shows that the transcriptomic switch that occurs in HC cows over the course of involution from an inflammatory to a restorative phenotype does not occur in cows that develop CE. qRT-PCR validation shows significantly increased inflammatory gene expression as well as the increased expression of genes encoding anti-inflammatory proteins, including IL-10, in cows with CE. By 21 DPP, the uterine environment in healthy cows has significantly reduced almost all inflammatory markers, and these signalling pathways have been replaced by genes involved in calcium regulation. The elevation of the genes encoding SERCA pumps, members of the solute carrier family and ryanodine receptor (*RYR*), calmodulin (*CaM*) and Ca2þ/calmodulin-dependent protein kinase II (*CaMKII*) that are increased highlighting an increased ability to regulate calcium levels and signalling in the uterus [39]. Calcium influx through the plasma membrane is also permitted by voltage-gated/operated/dependent calcium channels which are complexes containing several subunits (α_1_, α_2_δ, β_1–4_ and γ), the genes for which are also elevated at 21 DPP. Genes encoding important components that link this change in calcium concentration and prostaglandin F_2α_ (PGF) synthesis, [Phospholipases (*PLA2G3, PLA2G4B, PLCB1*); Protein kinases (*PRKCB, PRKCDBP* and *PRKAG2*) and prostaglandin F_2α_ synthase enzymes (*FAM213B)*] are significantly elevated in HC 21 DPP in this study. As PGF is a vital component of the estrus cycle [40], a greater capacity to resolve endometrial inflammation by 21 DPP may enhance a cow’s ability to elevate the production and secretion of PGF, permitting uterine tissue remodelling and normal cyclic progression compared to a cow experiencing sustained endometrial inflammation 21 DPP. The small numbers of differentially expressed genes between 7 and 21 DPP in the CE cows show that this transition did not occur and that inflammatory cell signalling was maintained.

MiRNA profiling may shed light on the regulation of the postpartum immune response and importantly, our results suggests that specific miRNAs may hold prognostic potential for endometritis. In the current study, all 5 members of the mir-200 family, *mir-200a*, *mir-200b*, *mir-200c*, *mir-141* and *mir-429*, are significantly elevated 7 DPP in CE cows relative to 21 DPP. The high differential expression of 14 miRNAs, including some of these members—specifically *mir-200b* and *mir-200c* are also higher in CE than HC cows at 7 DPP. Relevantly, studies in the human endometrium have revealed that miRNA expression regulates cellular proliferation, migration and the miR-200 family members assist in these processes [41, 42]. The Let-7 family of miRNA has been implicated in the regulation of the inflammatory reponse, through mediation of IL-6 and IL-10 cytokines [43] and *bta-let-7c* is significantly elevated in CE cows at 7 DPP in this study*.* Further investigation is required to determine the consequences of the significant increased regulation of this and additional miRNAs in CE cows at 7 DPP on the development of a restorative immune response within the uterus and their potential utilty as diagnostics of disease.

Following a normal parturition, consistent bacterial clearance and recontamination can occur within the first two weeks postpartum and in the absence of clinical illness, spontaneously clears by 10 to 15 DPP [44, 45]. Multiple bacterial species have been associated with the development of disease in the postpartum period including *Trueperella pyogenes* and *Escherichia coli* [44]. Emerging culture-independent techniques are providing unparalled resolution into the diversity of bacterial species present in the postpartum uterus and those potentially contributing to disease [10, 11]. Culture-independent analysis used in the current study shows significantly different microbial populations in the uterus of CE cows, which concurs with the differential expression of diverse TLRs. It is likely therefore that the divergent immune and restorative signalling pathways in the endometrium result, at least in part, from the composition of the microbial populations present.

The differential expression of Antimicrobial Peptides (AMPs) between HC and CE cows is of particular relevance to this study, especially given the shift in bacterial populations detected between groups. β-defensins are critical effector molecules of the immune response, which have been shown to be expressed in response to LPS in uterine epithelial cells [46]. Interestingly, genes encoding β-defensin AMPs (*DEFB5*, *DEFB7, DEFB405* and *LAP*) are significantly and elevated in expression in CE animals at 21 DPP (relative to HC), suggesting an inability to control infection. Furthermore, the S100 family of AMPs are LPS responsive [47] calcium-binding proteins [48] which have a wide variety of actions in innate immunity including the blocking of neutrophil migration [49] are also increased in CE samples at 21 DPP. *S100A14* has previously been reported as significantly increased in the endometrium from cows with endometritis [50]. The significant reduction in the expression of a number of β-defensin and S100 AMP genes by 21 DPP in HC cows only, suggests that CE cows have on-going infection.

An intricate relationship exists between metabolism and immunity [51] and central to both is the hepatic function [52]. As the main centre for mobilization of nutrients including non-esterified fatty acids (NEFAs) in addition to homeostatic and immune functions, which includes the synthesis of acute phase proteins, the liver is often used as a sentinel of physiological pressure, especially in the high-yielding dairy cow [53]. During severe negative energy balance, systemic concentrations of NEFAs and β-hydroxybutyrate (BHB) are elevated, indicating lipid mobilization and fatty acid oxidation [54] and circulating levels of glucose decrease [55]. In the current study HC and CE cows had peripheral concentrations of NEFAs and BHB below and glucose levels above those observed in severe NEB [55] and additionally, no significant differences in any of the blood metabolites analysed within and between groups 7 and 21 DPP was detected. Therefore we conclude that it is unlikely that NEB is contributing to the divergent immune response profiles detected between CE and HC cows in this study.

Although the link between inflammation, liver function and fertility are poorly understood [56], acute phase proteins are often used as an indicator of liver malfunctions across a range of species. It was of interest to note that many of the differentially expressed genes are classically thought to be of hepatic origin (APPs, transferrin and genes encoding complement factors) are also expressed in endometrial tissue. The divergence in APPs, particularly in HP expression levels between cows with uterine infection and cows that are healthy postpartum has been previously reported [57]. At 7 DPP, elevated HP has been associated with poorer fertility and avoiding the acute phase response during the transition period has been suggested as a possible route toward improving reproductive performance in high-yielding dairy cows [56]. While SAA levels are very dynamic during the peri-partum period, elevated expression levels of both HP and SAA have been proposed as potential diagnostics—particularly for clinical disease (metritis) [58, 59]. The concentrations of APP have been shown to reflect the magnitude of LPS exposure [60], suggesting that the concentration of uterine microbes will be reflected in APP expression levels. While there is no doubt that they are sensitive indicators of infection, the increased plasma expression detected at 7 DPP in CE cows in this study are unlikely to be specific for uterine infection as elevated HP and SAA have been shown in sera from mastitic cows experimentally infected with *S. aureus* [61]. However, the magnitude of the increased HP and SAA detected in this study is significantly higher than the levels found in cows with natural mastitis infection (median levels of 0.74 mg/ml and 29.9 μg/ml for HP and SAA, respectively) [62].

Other systemic immune changes detected in cows with CE was an early significant reduction in the numbers of circulating granulocytes—both neutrophils and eosinophils. It is presumed that this reduction in granulocyte number systemically reflects homing of these cells to the inflamed uterus as was detected histologically. Neutrophils and eosinophils use the molecules (including antimicrobial peptides) stored in their granules to kill microorganisms and they also produce reactive oxygen species (ROS) required for the formation of DNA-containing extra-cellular traps, which bind and kill bacteria. However eosinophils can modulate innate and adaptive immunity by producing immunoregulatory cytokines and are also involved in tissue repair and remodelling [63].

There is no doubt that inflammation is classically described as pathological, being the cause of significant morbidity and mortality. Multiple studies, and our earlier work has documented significantly elevated proinflammatory immune gene expression in the postpartum uterus [6, 21, 29]. However, the presence of inflammation is not necessarily detrimental; indeed a pro-inflammatory immune response has been shown to be beneficial to a successful pregnancy [64]. In humans, it has long been recognised that it is poor regulation of inflammation that contributes to poorer reproductive outcomes [65, 66]. In earlier work, the activation of inflammation and subsequent restoration of physiological function in the uterus of healthy cows by 30 DPP led us to conclude that postpartum inflammation is a normal physiological event [3]. Significantly, we then identified a temporal switch that healthy animals undergo from a proinflammatory gene expression profile to a regenerative and proliferation profile [22]. The current study supports this trend and shows that in animals which develop CE, this transition is arrested or delayed leading to sub-optimal restoration of homeostasis which is likely to have negative reproductive outcomes. In that regard, manipulating the expression of antimicrobial peptides (β-defensin and S100A) and immunoregulatory molecules (including IL-10) may hold the key to regulating inflammation and the restoration of homeostasis in the bovine uterus, thereby improving disease outcomes.

Although there is no doubt that the development of endometritis results from complex interplay between microbes and host factors, analysis of the uterine transcriptional landscape, at both miRNA and mRNA levels, has provided a powerful insight into the regulation of immunity during the critical peri-parturient period. While reliable information on the role and gene targets of miRNAs is currently limited, particularly in cattle, the analysis of specific miRNAs identified as differentially expressed in this study can now be targeted in follow on functional studies. This combined dataset complements profiling at a systemic level, suggesting that even in cows with SCE, early changes in peripheral blood can be detected. While this study is limited by the numbers of infected cattle analysed at only two post-partum time-points, a number of novel hypotheses have been generated that can now be tested in larger numbers of post-partum cattle with endometritis.

## Conclusions

Although it is recognised that the development of endometritis results from complex interplay between microbes and host factors, analysis of the uterine transcriptional landscape, at both miRNA and mRNA levels, has provided a powerful insight into the regulation of immunity during the critical peri-parturient period. While reliable information on the role and gene targets of miRNAs is currently limited, particularly in cattle, the analysis of specific miRNAs identified as differentially expressed in this study can now be targeted in follow on functional studies. This combined dataset complements profiling at a systemic level, suggesting that even in cows with SCE, early changes in peripheral blood can differentiate them from healthy cows. While this study is limited by the numbers of infected cattle analysed at only two post-partum time-points, a number of novel hypotheses have been generated that can now be tested in larger numbers of post-partum cattle with endometritis.

## Methods

### Animals and sample collection

Fifteen Holstein-Friesian cows, of mixed parity, within the same university dairy herd were sampled 7 and 21 days postpartum (DPP) in the morning after milking. A clinical examination and sample collection were conducted by a veterinarian and involved recording the rectal temperature, body condition score, heart rate and vaginal discharge. At each time-point, an endometrial biopsy was taken from the same post-gravid horn as previously described [67]. Uterine culture swabs were placed (in duplicate per cow) in 1 ml of Tris-EDTA buffer (Sigma Aldrich® Vale Road, Arklow, Wicklow, Ireland). Immediately after collection, the biopsy was dissected in two - one half was snap frozen in liquid nitrogen for transcriptomic analysis, and the other half was fixed in 10 % neutral-buffered formalin solution (Sigma Aldrich® Vale Road, Arklow, Wicklow, Ireland) for histopathological assessment. Whole blood was collected in spray-coated K_2_EDTA Vacutainer®, for haematology and plasma protein analysis, followed by 4 ml of blood in one sodium fluoride/Na_2_ EDTA Vacutainer®, for blood metabolite analysis at pre- and postpartum time points. All experimental procedures were carried out under license from the Irish Department of Health and Children in accordance with the European Community Directive 86-609-EC and were approved by the Animal Research Ethics Committee, University College Dublin.

### Histopathology

Formalin fixed uterine tissues at each time point were paraffin-embedded, sectioned at 5–8 μm thickness, mounted on glass slides and stained with haematoxylin and eosin (H&E). The degree of inflammation in endometrial biopsies was graded blind without knowledge of sample ID or background. Endometrial inflammation was classified based on previous criteria outlined by Chapwanya et al. [67], which focused on intensity of inflammatory cell infiltration of the stroma, in combination with a study by Meira et al. [68], which incorporated the numbers of polymorphonuclear neutrophils within the endometrial epithelium. Each section was graded firstly on the intensity of epithelial polymorphonuclear (PMN) infiltrate and secondly on the intensity of stromal inflammatory cell infiltration, the greater the number of PMN in the epithelium the higher the score which ranged between 0 and 3 (Fig. 1).

### Haematology

Total red blood cell, neutrophil, lymphocyte, monocyte, eosinophil and basophil cell counts were determined from K_2_EDTA anti-coagulated blood with an automated haematology analyser (ADVIA 2120, Bayer Healthcare, Siemens, UK).

### RNA extraction, library preparation and NGS sequencing

A Pro Scientific homogeniser model PRO200 (PRO Scientific Inc., Oxford, CT, USA) was used to homogenise the biopsy in TRIzol® (Life Technologies™, Paisley, UK) with 3 s pulses 10 times. Total RNA was then cleaned up using the Qiagen RNeasy® Plus Mini kit (Qiagen Ltd). The Nano-Drop ND-1000 UV–vis Spectrophotometer (NanoDrop Technologies Inc., Wilmington, DE, USA) was used to quantify the concentration of RNA. The Agilent 2100 Bioanalyser (Agilent Technologies) was used to assess the quality of RNA, and RIN values ranged from 6 to 9.9 across all samples used (Additional file 1: Table S1). The Illumina® TruSeq® RNA sample preparation kit v2 (Chesterford Research Park, Little Chesterford, Nr Saffron Walden, Essex, CB10 1XL, UK) was used to convert mRNA into cDNA libraries for DNA sequencing. Input total RNA was uniformly 4 μg in a volume of 50 μl nuclease-free H_2_O for each sample. Indexes from Illumina® TruSeq® v2 A and B kits were allocated to specific samples prior to library construction so that at least two unique and compatible bar codes were in each pool which made provision for a pooling strategy following the Illumina® TruSeq® pooling guidelines. After adapter ligation the ds cDNA fragments were enriched by PCR which created the final sequencing cDNA library. In the PCR enrichment stage of the protocol 10 PCR cycles were used. The Agilent 2100 Bioanalyser (Agilent Technologies) was used to assess the purity of the ds cDNA libraries using a DNA chip protocol. Seven equimolar pools of 5 multiplexed cDNA sample libraries were constructed. Indexes from Illumina® TruSeq® v2 kits A and B were allocated to specific samples following the Illumina® TruSeq® pooling guidelines. The sequencing of 50 bp reads paired-end cDNA libraries was performed by the Beijing Genomics Institute (BGI) (BGI Hong Kong Co., Limited, Tai Po, New Territories, Hong Kong) using an Illumina® HiSeq™ 2000.

### mRNA-Seq data analysis

Quality control checks of 49 bp paired-end sequencing reads was performed using FastQC software (version 0.10.0) [http://www.bioinformatics.babraham.ac.uk/projects/fastqc/]. All reads were above a Phred quality score threshold of 20. Prior to aligning sequences to the genome, TopHat (version 2.0.6) was used to estimate the inner mate distance between paired reads for each library. Paired reads were aligned to the bovine genome (UMD 3.1.69) with Bowtie2 (version 2.0.5), using a maximum fragment length (−X) of 500 and minimum fragment length (−I) of 0 for valid paired-end alignments. The output files were sorted using the picard-tools (version 1.60) SortSam option using the sort order command “coordinate” and Picard-tools CollectInsertSizeMetrics option. Only reads that mapped uniquely to the bovine genome were kept (−g 1). The default value of two mismatches per read was allowed. Sensitivity of Bowtie2 was set for the best alignment of each read (−−b2-very-sensitive). Only concordantly mapped reads were allowed (−−no-disconcordant), and paired reads that didn’t map concordantly were not aligned separately (−−no-mixed). The segment length was set to 24 bp (−−segment-length 24). HTSeq-count, (version 0.5.3p9, http://www-huber.embl.de/users/anders/HTSeq/doc/overview.html) utilising default parameters, with the exception of specifying non strand-specific data (−s no), assigned uniquely aligned reads to the Ensembl (version 69) annotation of the bovine genome. Bioconductor package edgeR (edgeR_3.2.4) [69] was applied in R (version 3.0.1) to identify statistically significant differentially expressed genes. To account for biological and technical variation, data was modeled as a negative binomial distribution using a generalisation of the Poisson distribution model. A low read count filter was applied to remove lowly expressed genes. Genes with greater than 1 read count per million reads (cpm), in n-1 animals, were retained. The data was normalised across library sizes, between samples using the trimmed mean of M-values (TMM) normalization method [70]. Tagwise dispersions were estimated for the normalised dataset. Statistical tests were corrected for multiple testing using the Benjamini-Hochberg method. Genes that were differentially expressed (DE) with a false discovery rate (FDR) adjusted *P*-value < 0.1 were retained. Summary statistics for the mRNA data is given in Additional file 1: Table S1. A full list of DEG is given in Additional file 2: Table S2. Heatmaps of the 30 most significantly differentially expressed genes was generated in R using the heatmap.2 function in the ggplots CRAN library.

### Gene ontology over-representation analysis

Bovine gene IDs retained with an adjusted *P*-value < 0.1, were converted to human orthologs using the BioMart application on the Ensembl website (http://www.ensembl.org/biomart/martview/). GOseq (version 1.18.0) was implemented in R (version 3.1.1) to identify significantly over-represented Gene Ontology terms, in the dataset of DE genes. Goseq performs gene legth bias correction by obtaining UCSC gene length data and GO mappings from organism packages and calculates the probability weighting function (PWF), to account for gene length bias. *P*-values were determined by the GOseq “Wallenius” default method [71].

### miRNA-Seq data analysis

Adapter sequences were trimmed with Cutadapt (version 1.1) (http://www.cutadapt/). Reads shorter than 18 bp were discarded, and the fastq quality filter (http://hannonlab.cshl.edu/ fastx_toolkit/) (version 0.0.13) removed reads where less than 50 % of bases with a Phred score of 20. Reads passing all the filters were also trimmed at their ends to remove low-quality bases (Phred score < 20). High quality reads were aligned to the bovine genome (UMD 3.1) with Novoalign (version 2.07.11) (http://www.novocraft.com) implementing the “-m” miRNA mode. Reads that aligned to more than one position in the genome were discarded. HTSeq-count (version 0.5.3p9, http://www-huber.embl.de/users/anders/HTSeq/doc/overview.html) default parameters were used, with the exception of specifying non strand-specific data (−s no), to assign uniquely aligned reads to the Ensembl (version 66) annotation of the bovine genome. miRNA-Seq differential expression analysis was applied with the same parameters as described above for mRNA-Seq differential expression analysis.

### Quantitative real-time PCR validation

The reverse transciption for real-time PCR was performed as previously described [22] using uterine biopsy RNA (converted to cDNA) from the same group of cows used in the generation of the NGS data (except one SCE cow where insufficient RNA was available). Fifteen genes were selected for validation of the NGS results based on a combination of criteria including an adjusted *P* < 0.1, RPKM values and fold change. Intron-spanning, gene-specific primers were designed using the primer-blast tool on the NCBI website (www.ncbi.nlm.nih.gov/tools/primer-blast) and are listed in Table S7. Reference genes were analysed from the NGS data and *PPIA* identified as non-differentially expressed and therefore most stable for normalisation. Relative gene expression levels were calculated using the 2^-ΔΔCt^ method [72] and significant differences were identified using a Student’s t-test on normalised Ct values.

### Plasma protein quantitation

Plasma was analysed for the presence of acute phase proteins (APPs) serum amyloid A (SAA) and haptoglobin (HP). SAA was measured using the PHASE™ Serum Amyloid A Assay (SAA) kit, a solid phase sandwich Enzyme Linked Immuno Sorbent Assay (ELISA), following the manufacturer’s protocol and was supplied by Tridelta Development Ltd, Maynooth, Co. Kildare, Ireland. The intra-assay coefficient of variation for serum SAA was 5.5 %. The limit of detection for this assay was 18 mg/ml. HP was measured using the Tridelta PHASE™ Haptoglobin Assay kit, a colorimetric assay, following the manufacturer’s protocol (supplied by Tridelta Development Ltd, Maynooth, Co. Kildare, Ireland). The intra-assay coefficient of variation for serum HP was 4.3 %. The limit of detection for this assay was 312 ug/ml. Sample absorbances were read using the GloMax®-Multi Detection System from Promega. ELISAs performed using commercially available kits for bovine IL-1β (IL-1 beta ELISA Reagent Kit, Bovine) and bovine IL-6 (IL-6 ELISA Reagent Kit, Bovine, ThermoScientific®, 3747 N. Meridian Rd. Rockford, IL 61101, United States). The intra-assay coefficient of variation for serum IL-6 was 3.8 %. The limit of detection for this assay was 0.8 ng/ml. For IL-8, a commercially available human IL-8 ELISA kit (R&D Systems Inc., Minneapolis, Minnesota, USA) was used to measure bovine IL-8 according to the manufacturer’s instructions. The antibodies used in the kit have been previously validated to cross-react with bovine IL-8 [73]. IL-17A was also measured using a commercial ELISA (bovine IL-17A ELISA Reagent Set, GenWay Biotech, Inc 6777 Nancy Ridge Drive, San Diego, CA 92121, USA).

### Metabolite analysis

Plasma was analysed to quantify levels of blood metabolites including non-esterified fatty acids (NEFA) [Randox Laboratories, Belfast, Northern Ireland], β-hydroxybutyrate (BHB) [Randox Laboratories, Belfast, Northern Ireland], glucose and urea (Audit Diagnostics, Cork, Ireland) and was performed on Beckman Coulter AU 400 clinical analyser using test protocol supplied by the manufacturer. Glucose and Urea tests were calibrated using a Beckman Coulter multi-calibrator with levels for each metabolite traceable to NIST standard reference material. BHB and NEFA calibrators are supplied with each kit. Quality control samples (Tri-level) used are commercially available bovine specimens and CV for each metabolite tested was <6 %.

#### Statistics for haematology, APP and metabolite analysis

Initially a Kruskal-Wallis test, a non-parametric one way analysis of variance (ANOVA), was performed comparing pre-calving (*n* = 8), CE (*n* = 5) cows at 7 and 21 DPP and HC (*n* = 9) cows at 7 and 21 DPP. Subsequently, under the assumptions of non-normality, a Mann-Whitney test was used to determine significance between groups and a Wilcoxon paired test was used to determine significance over time.

### Microbiology

Terminal-restriction fragment length polymorphism (T-RFLP) was used to obtain fingerprints of the microbial communities associated with 28 duplicate endometrial swab samples collected at 7 and 21 DPP from 14 cows. DNA was extracted using the DNeasy Blood and Tissue following manufacturer’s directions for Gram positive bacteria (Qiagen Ltd., Crawley, UK). Briefly, pellets were suspended in 180 μl TE buffer supplemented with 0.2 % Triton X-100 and 20 mg/ml egg white lysozyme and incubated at 37 °C for 2 h. Proteinase K digestion of the sample was performed at 56 °C for 1 h. A further incubation at 90 °C for 5 min was included to help degrade the bacterial cell wall. AL buffer was added to the samples and loaded on the column. DNA was eluted in 50 μl. Extracted DNA was stored at −80 °C until further use. Amplification of the 16S rRNA genes was performed by nested PCR. First, a ~1.5 kb fragment was amplified (15 cycles) using primers 27 F-CM (5′-AGAGTTTGATCMTGGCTCAG) and 1492R (5′- TACGGYTACCTTGTTACGACTT) [74, 75]. The resulting amplicon was used as template for the generation of a ~1 kb fluorescently-labelled product using the 6FAM-27 F and U1052R (5′- GARCTGRCGRCRRCCATGCA) primers. Purified DNA was digested with *Msp*I following the manufacturer’s recommendations (New England Biolabs). DNA was then ethanol precipitated and resuspended in Hi-Di Formamide (final concentration 50 ng/μl) containing GeneScan-500 LIZ Size Standard (Applied Biosystems). Fragments were separated by capillary electrophoresis using a 3130*xl* capillary array (36 cm) in an ABI 3130*xl* Genetic Analyzer (Applied Biosystems). Fragment sizes were determined using GeneMapper v4.0 (Applied Biosystems). Merging of biological replicates and multiple alignment of T-RFLP profiles was performed with T-Align [76]. Only fragments present in both biological replicates and contributed at least 1 % of the total fluorescence signal were included in the analysis. Principal Coordinates Ordination (PCO) and Permanova analysis (999 permutations) were performed in Primer6 v6.1.13 and Permanova + v1.0.3 [77] based on the S17 Bray-Curtis similarity using square root transformed relative abundances obtained from the fluorescent signal of terminal restriction fragments.

### Additional material

The data discussed in this publication have been deposited in NCBI’s Gene Expression Omnibus [78] and are accessible through GEO Series accession number GSE66827 (http://www.ncbi.nlm.nih.gov/geo/query/acc.cgi?acc=GSE66827).

## Additional files

Additional file 1: Summary statistics for 30 mRNA and 20 miRNA libraries as well as RNA quality information.(XLSX 20 kb) Additional file 2: All differentially expressed gene lists for each comparison shown in Figure 2a (FDR < 0.1).(XLSX 987 kb) Additional file 3: KEGG pathway analysis of differentially expressed genes (FDR < 0.1).(XLSX 45 kb) Additional file 4: Significantly enriched GO terms in differentially expressed gene sets.(XLSX 1360 kb) Additional file 5: All differentially expressed miRNA lists for each comparison shown in Figure 2a (FDR < 0.1).(XLSX 88 kb) Additional file 6: Table S6. Significantly increased immune gene expression (Log2 FC >1.5, FDR >0.1) in CE samples at 21 DPP (DOCX 18 kb) Additional file 7: Table S7. Oligonucleotide primer sequences used for qRT-PCR validation. (DOCX 17 kb)
