# Erratum to: ‘Integrated analysis of the local and systemic changes preceding the development of post-partum cytological endometritis’

**DOI:** 10.1186/s12864-015-2205-x

**Published:** 2015-12-10

**Authors:** Cathriona Foley, Aspinas Chapwanya, John J. Callanan, Ronan Whiston, Raúl Miranda-CasoLuengo, Junnan Lu, Wim G. Meijer, David J. Lynn, Cliona O’Farrelly, Kieran G. Meade

**Affiliations:** Animal & Bioscience Research Department, Animal & Grassland Research and Innovation Centre, Teagasc, Grange, Co., Meath, Ireland; Comparative Immunology Group, School of Biochemistry and Immunology, Trinity College, Dublin 2, Ireland; Ross University, School of Veterinary Medicine, St Kitts, Basseterre, West Indies Dominica; UCD School of Veterinary Medicine, University College Dublin, Dublin 4, Ireland; UCD School of Biomolecular and Biomedical Science and UCD Conway Institute of Biomolecular and Biomedical Research, University College Dublin, Dublin 4, Ireland; South Australian Health & Medical Research Institute, North Terrace, Adelaide, 5000, SA Australia; School of Medicine, Flinders University, Bedford Park, Flinders, 5042, SA Australia

Unfortunately, the original version of this article [1] contained an error. The figure legends did not correspond to the correct figures, the correct version can be seen below.

**Fig. 3:**
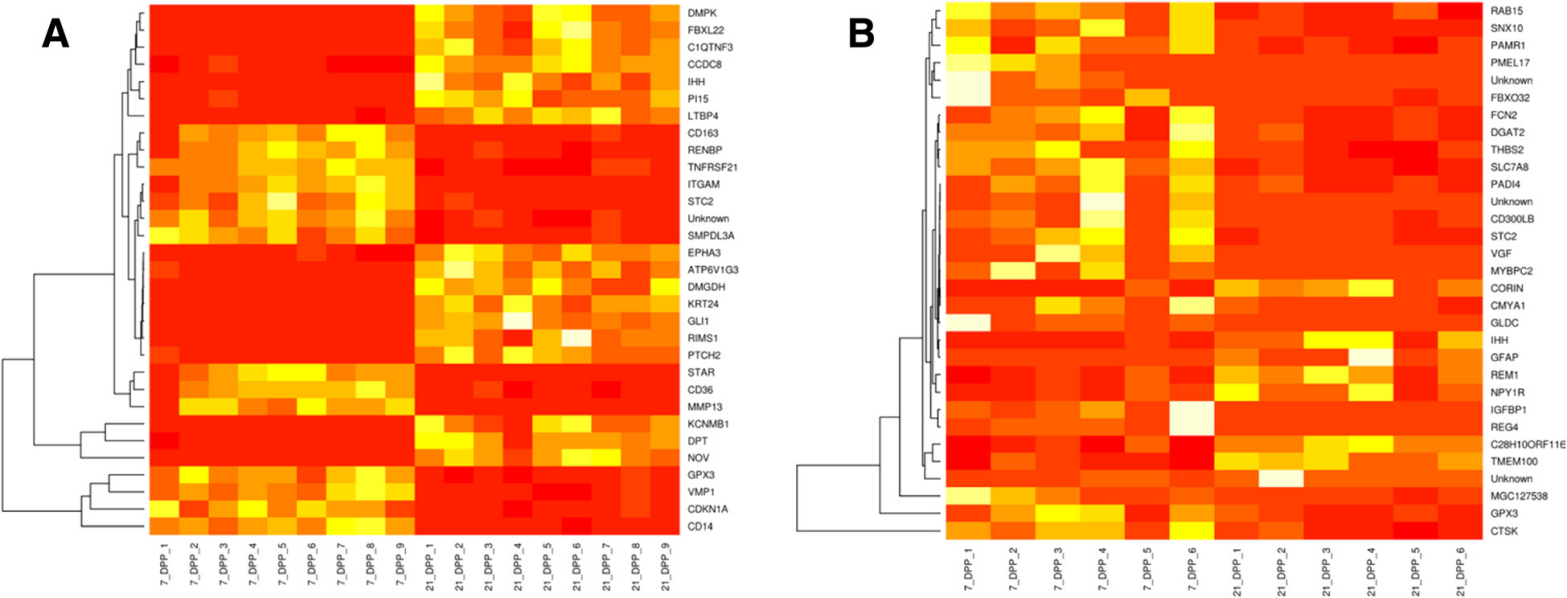
Heat map representation depicting the temporal changes in significantly differentially expressed genes from uterine biopsies between 7 and 21 DPP in the (**a**) HC and (**b**) CE cows. All 31 significantly DEGs were used to generate the heatmap in the CE group, and a similar number of the top DEGs (ranked on basis of *P* value) were used for comparative purposes for the HC group. Scale: Yellow indicates high expression and red is low expression. Unsupervised hierarchical clustering dendograms are included for these genes. **a** – DE genes between day 7 and 21 DPP in HC animals and (**b**) – DE genes between day 7 and 21 DPP in CE cows

**Fig. 5:**
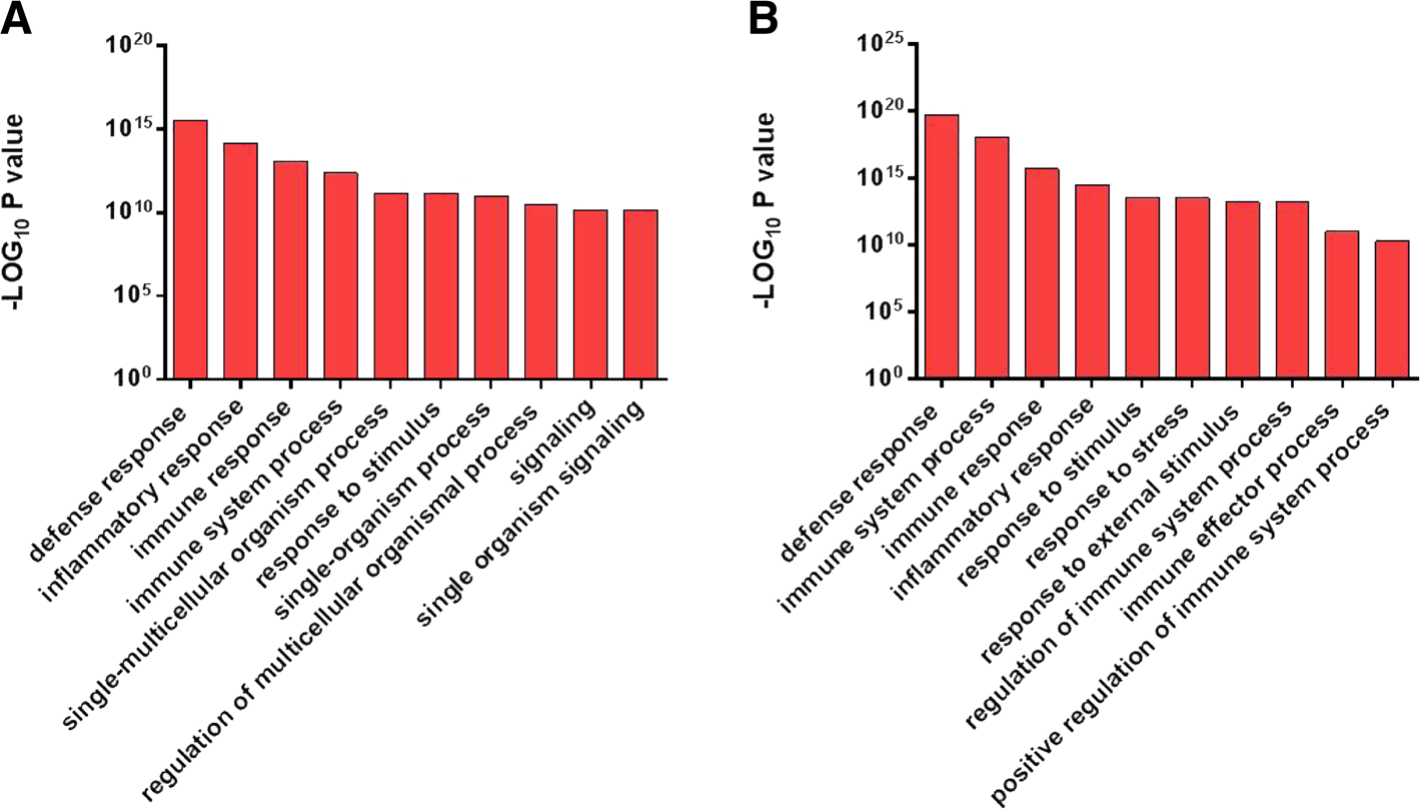
Top 10 Significantly Enriched Biological Processes in the Endometrium Identified by Gene Ontological Analysis. Using significantly differentially expressed gene datasets, gene ontology analysis identifed the enriched biological processes (**a**) in HC cows between 7 and 21 DPP and (**b**) between HC and CE samples at 21 DPP. The resolution of the inflammatory response in HC cows (**a**) is evident as these defence and innate immune response processes are switched off. The sustained inflammatory response (lack of transition) is evident in (**b**) as these processes are enriched in CE compared to HC at 21 DPP. For full list of enriched gene ontologies, see Table S4

**Fig. 6:**
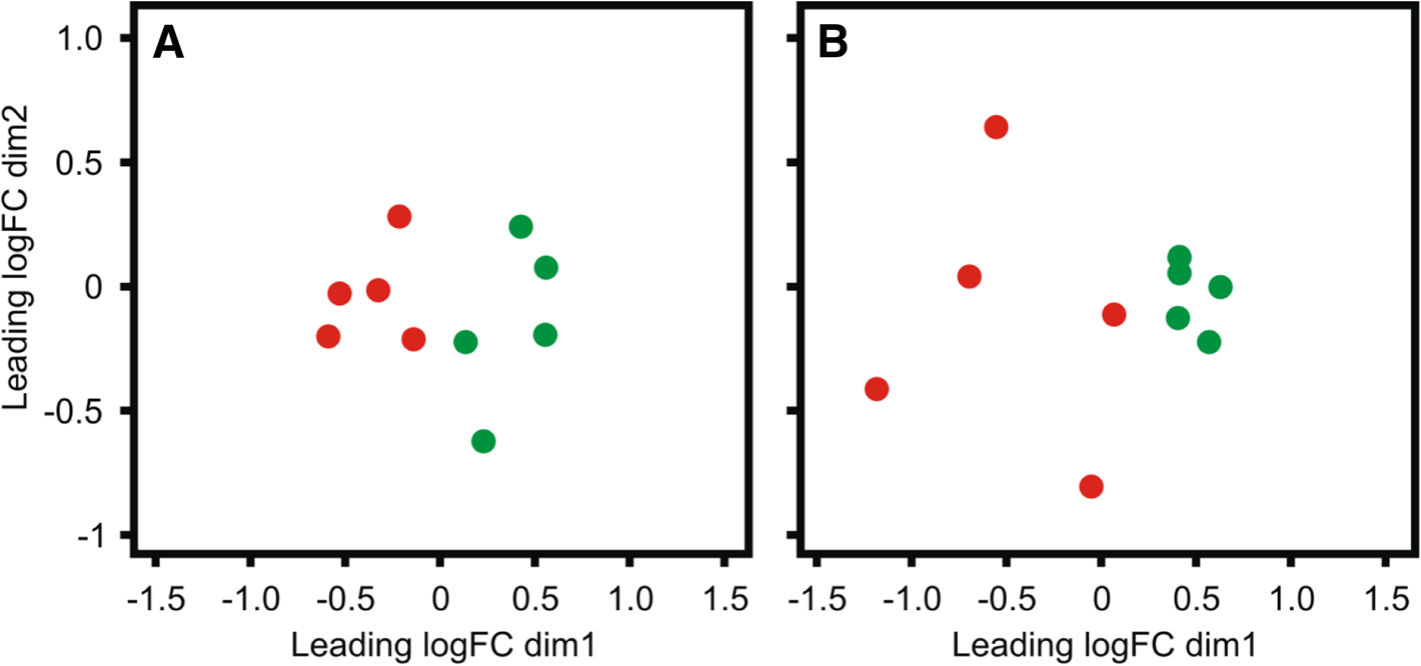
Multi-Dimensional Scaling (MDS) Plots Generated from Endometrial microRNA-seq Data. **a** MDS-plot shown for HC cows at both 7 (red) and 21 DPP (green). Similarly, (**b**) MDS-plot shown for CE cows at both 7 (red) and 21 DPP (green). Clustering of the D7 and D21 profiles is apparent at both time points for HC and CE cows although tighter clustering of 7 DPP samples for the HC group than for the CE group (B) shows a higher degree of variation between samples at 7 DPP (B, red). Five HC (healthy control) samples at both 7 DPP (D7, red) and corresponding same animal sample at 21 DPP (D21, green) and B) five CE (cytologically endometritic) cows at the same two time points are shown

**Fig. 7:**
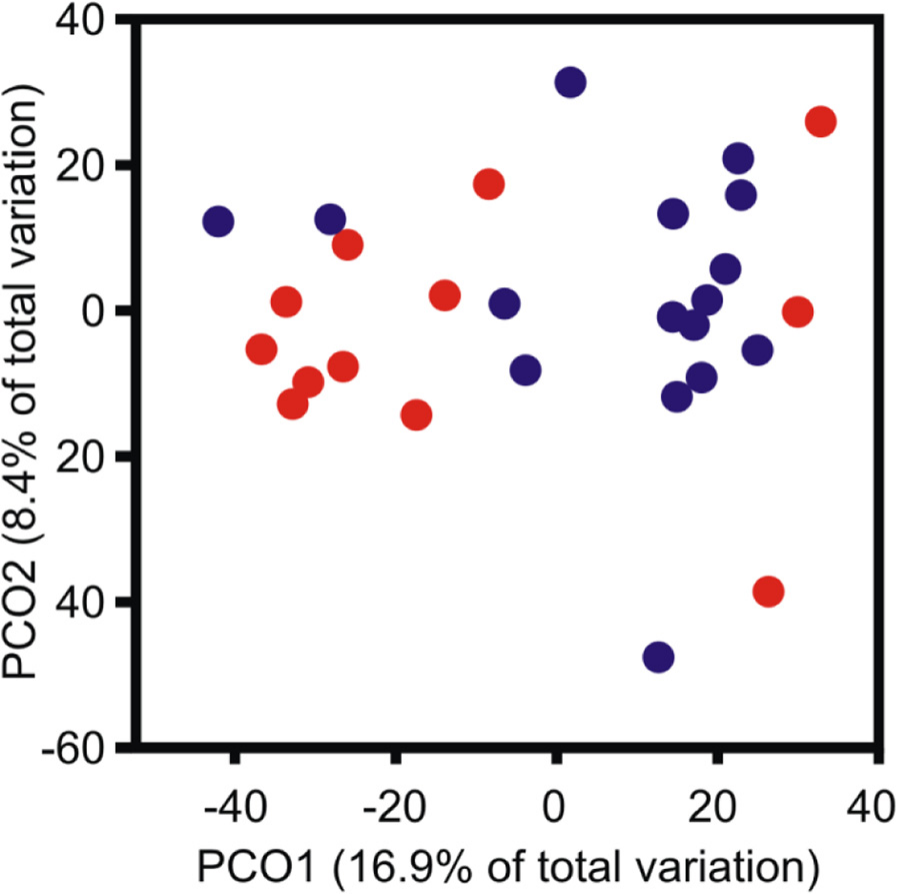
Principal Coordinates Ordination of Endometrial Bacterial Communities. Bacterial community analysis, performed using culture-independent Terminal Restriction Fragment Length Polymorphism (T-RFLP), shows a significant clustering of samples according to their respective microbial communities. HC and CE cows are shown as blue and red circles, respectively

**Fig. 8:**
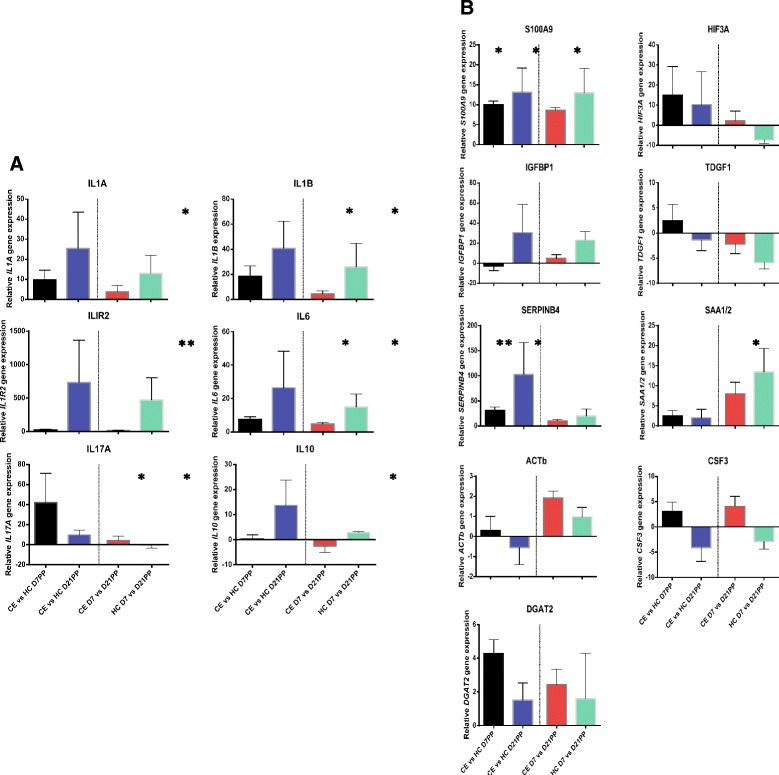
RT-qPCR validation of gene expression changes detected using mRNA-seq. Confirmation of differentially expressed genes from NGS results using quantitative real-time PCR. Significant changes in gene expression of both (**a**) pro- and anti-inflammatory cytokines as well as other (**b**) effector molecules of the immune response confirmed the findings from NGS. Results are colour-coded according to comparison and levels of expression of each gene of interest was normalised to expression levels of *PPIA*; D7PP between SCE and HC cows (black), D21PP between SCE and HC (blue), between D7 and D21PP in SCE (red) and between D7 and D21PP in HC (green). Between and within group comparisons are separated by a dotted line. *P < 0.05; **P < 0.01. *n* = 5-8 samples per time point, bars represent mean ± SEM
