# Supplementary material for: Integrated analysis of the local and systemic changes preceding the development of post-partum cytological endometritis
Source: BMC Genomics. 2015 Oct 19;16:811. doi: 10.1186/s12864-015-1967-5 (PMC4617749; doi:10.1186/s12864-015-1967-5)
Supplement: Additional file 6: Table S6. — Significantly increased immune gene expression (Log2 FC >1.5, FDR >0.1) in CE samples at 21 DPP (DOCX 18 kb) [file 12864_2015_1967_MOESM6_ESM.docx]

Table S6: Significantly increased immune gene expression (Log_2_ FC >1.5, FDR >0.1) in CE samples at 21 DPP

| **Gene symbol** | **Ensembl ID** | **Gene name** | **log_2_ FC** | **FDR** |
| --- | --- | --- | --- | --- |
| Cell surface receptors | | | | |
| *CD27* | ENSBTAG00000014725 | CD27 molecule | 2.47 | 0.000181 |
| *CD38* | ENSBTAG00000013569 | CD38 molecule | 1.99 | 0.004573 |
| *CD48* | ENSBTAG00000011238 | CD48 molecule | 1.61 | 0.000927 |
| *CD79A* | ENSBTAG00000001882 | CD79A antigen (immunoglobulin-associated alpha) | 2.46 | 0.000761 |
| *CD79B* | ENSBTAG00000044204 | CD79A antigen (immunoglobulin-associated beta) | 2.87 | 0.001189 |
| *CD80* | ENSBTAG00000018059 | CD80 molecule | 1.89 | 0.004777 |
| Chemokine ligands and receptors | | | | |
| *CCL20* | ENSBTAG00000021326 | Chemokine (C-C motif) ligand 20 | 2.84 | 0.003152 |
| *CCL22* | ENSBTAG00000017718 | Chemokine (C-C motif) ligand 22 | 1.92 | 0.017947 |
| *CCL24* | ENSBTAG00000026275 | Chemokine (C-C motif) ligand 24 | 1.90 | 0.074332 |
| *CXCL3* | ENSBTAG00000037778 | Chemokine (C-X-C motif) ligand 3 | 1.82 | 0.004449 |
| *CXCL6* | ENSBTAG00000009812 | Chemokine (C-X-C motif) ligand 6 | 3.20 | 4.18E-05 |
| *CXCL14* | ENSBTAG00000006694 | Chemokine (C-X-C motif) ligand 14 | 1.82 | 0.001391 |
| *CXCL17* | ENSBTAG00000018652 | Chemokine (C-X-C motif) ligand 17 | 3.02 | 0.037411 |
| *CXCR2* | ENSBTAG00000026753 | Chemokine (C-X-C motif) receptor 2 | 2.34 | 0.067343 |
| Cytokines and receptors | | | | |
| *IL1R2* | ENSBTAG00000006343 | Interleukin 1 receptor, type II | 5.99 | 1.38E-07 |
| *IL6* | ENSBTAG00000014921 | Interleukin 6 | 4.59 | 0.000212 |
| *IL10* | ENSBTAG00000006685 | Interleukin 10 | 1.66 | 0.015902 |
| *IL11* | ENSBTAG00000047400 | Interleukin 11 | 3.30 | 6.15E-05 |
| *IL18RAP* | ENSBTAG00000033748 | Interleukin 18 receptor accessory protein | 1.62 | 0.01238 |
| *IL20RB* | ENSBTAG00000008299 | Interleukin 20 receptor beta | 1.77 | 0.012088 |
| *IL21R* | ENSBTAG00000019567 | Interleukin 21 receptor | 1.33 | 0.013131 |
| *Antimicrobial peptides* | | | | |
| *DEFB5* | ENSBTAG00000034954 | Defensin, beta 5 | 1.58 | 0.080315 |
| *DEFB7* | ENSBTAG00000046144 | Defensin, beta 7 | 3.07 | 0.007787 |
| *DEFB405* | ENSBTAG00000035183 | Beta-defensin 10 | 3.80 | 2.33E-05 |
| *LAP* | ENSBTAG00000027225 | Lingual antimicrobial peptide | 3.07 | 0.020247 |
| *S100A2* | ENSBTAG00000037651 | S100 calcium binding protein A2 | 2.15 | 0.002316 |
| *S100A5* | ENSBTAG00000000644 | S100 calcium binding protein A5 | 2.89 | 2.61E-05 |
| *S100A8* | ENSBTAG00000012640 | S100 calcium binding protein A8 | 3.74 | 0.000896 |
| *S100A9* | ENSBTAG00000006505 | S100 calcium binding protein A9 | 3.92 | 0.000316 |
| *S100A12* | ENSBTAG00000012638 | S100 calcium binding protein A12 | 3.49 | 0.000847 |
