# Supplementary material for: Integrated analysis of the local and systemic changes preceding the development of post-partum cytological endometritis
Source: BMC Genomics. 2015 Oct 19;16:811. doi: 10.1186/s12864-015-1967-5 (PMC4617749; doi:10.1186/s12864-015-1967-5)
Supplement: Additional file 7: Table S7. — Oligonucleotide primer sequences used for qRT-PCR validation. (DOCX 17 kb) [file 12864_2015_1967_MOESM7_ESM.docx]

Table S7: Oligonucleotide primer sequences used for qRT-PCR validation

| **Gene symbol** | **Ensembl ID** | **Forward primer (5’-3’)** | **Reverse primer (5’-3’)** |
| --- | --- | --- | --- |
| *IL1A* | ENSBTAG00000010349 | AGCCAGTGGGAAGATTCTGA | GCATTCCTGGTGGATGACTC |
| *IL1B* | ENSBTAG00000001321 | TCCACCTCCTCTCACAGGAAA | TACCCAAGGCCACAGGAA |
| *IL1R2* | ENSBTAG00000006343 | GGAGACAAGCTCCAGGCTCT | GAACCTTGTTTTGGGGTGCC |
| *IL6* | ENSBTAG00000014921 | CCAGAGAAAACCGAAGCTCTCAT | CCTTGCTGCTTTCACACTCATC |
| *IL17A* | ENSBTAG00000002150 | CGTTAACCGGAGCACAAACT | TCCCAGATCACAGAGGGGTA |
| *IL10* | ENSBTAG00000006685 | GAAGGACCAACTGCACAGCTT | AAAACTGGATCATTTCCGACAAG |
| *S100A9* | ENSBTAG00000006505 | GGGCACTATGACACCCTGAT | GTGTTGTGCATCTCCTCGTG |
| *HIF3A* | ENSBTAG00000018948 | AGTCATGGCGCTGGGACTAC | GGTAAGCCATGTCTCCCTCG |
| *IGFBP1* | ENSBTAG00000046768 | CAGCCCAGAGAATGTGTCCC | GTAGAGTTCTCGCTGGCAGG |
| *TDGF1* | ENSBTAG00000021119 | ATTGTTAGGGGCCACAGACC | GGAAGGCTGGTGACGAATTG |
| *SERPINB4* | ENSBTAG00000039037 | ATGCTGCAGAGGAAAGTCGA | GGTTCCACTGCCCTTTGAAG |
| *SAA1/2* | ENSBTAG00000022394 | CACAGGCCTCATTCTCTGCT | TCTGCACCCTTGTAGTTGGC |
| *ACTB* | ENSBTAG00000015441 | AGATGACCCAGATCATGTTCGA | TGACCCCGTCACCGGAGTCCATCACGAT |
| *CSF3* | ENSBTAG00000021462 | CTGAACCAACTACACGGCGG | CTCAGGGCTCAGCAAGGTAG |
| *DGAT2* | ENSBTAG00000001154 | AGTACATTGGCTTTGCCCCA | ACGTACATGGCGTGGTACAG |
| *PPIA* | ENSBTAG00000012003 | CCACCGTGTTCTTCGACAT | TCTGTGAAGCAGGAACCTTT |
|  |  |  |  |
